# Continuous protein-density gradients: A new approach to correlate physical cues with cell response

**DOI:** 10.1093/pnasnexus/pgae202

**Published:** 2024-05-21

**Authors:** Shanshan Zhang, Oliver Felthaus, Lukas Prantl, Nan Ma, Rainhard Machatschek

**Affiliations:** Institute of Chemistry and Biochemistry, Freie Universität Berlin, Takustrasse 3, 14195 Berlin, Germany; Helmholtz-Zentrum Hereon, Institute of Active Polymers, Kantstrasse 55, 14513 Teltow, Germany; Department of Plastic Surgery, University Hospital Regensburg, Franz-Josef-Strauss-Allee 11, 93053 Regensburg, Germany; Department of Plastic Surgery, University Hospital Regensburg, Franz-Josef-Strauss-Allee 11, 93053 Regensburg, Germany; Institute of Chemistry and Biochemistry, Freie Universität Berlin, Takustrasse 3, 14195 Berlin, Germany; Helmholtz-Zentrum Hereon, Institute of Sustainable Materials, Kantstrasse 55, 14513 Teltow, Germany; Helmholtz-Zentrum Hereon, Institute of Active Polymers, Kantstrasse 55, 14513 Teltow, Germany

**Keywords:** laminin, gradient, Langmuir–Blodgett, hADSC, single-cell force spectroscopy

## Abstract

To assess cellular behavior within heterogeneous tissues, such as bone, skin, and nerves, scaffolds with biophysical gradients are required to adequately replicate the in vivo interaction between cells and their native microenvironment. In this study, we introduce a strategy for depositing ultrathin films comprised of laminin-111 with precisely controlled biophysical gradients onto planar substrates using the Langmuir–Blodgett (LB) technique. The gradient is created by controlled desynchronization of the barrier compression and substrate withdrawal speed during the LB deposition process. Characterization of the films was performed using techniques such as atomic force microscopy and confocal fluorescence microscopy, enabling the comprehensive analysis of biophysical parameters along the gradient direction. Furthermore, human adipose-derived stem cells were seeded onto the gradient films to investigate the influence of protein density on cell attachment, showing that the distribution of the cells can be modulated by the arrangement of the laminin at the air–water interface. The presented approach not only allowed us to gain insights into the intricate interplay between biophysical cues and cell behavior within complex tissue environments, but it is also suited as a screening approach to determine optimal protein concentrations to achieve a target cellular output.

**Significance Statement**

The Langmuir–Blodgett technique is a versatile method to fabricate homogeneous mono- and multilayers of amphiphilic molecules and particles. In this work, the method is modified to fabricate protein-based ultrathin films with density gradients. Through the variation of the areal packing density, both the stiffness and the organization of the deposited laminin-111 change continuously along the gradient direction. Such films can be used as screening method to correlate the density of deposited proteins, the biophysical properties of the resulting layer and the response of cells trough a single experiment. The approach is validated for two types of cells, both of which show an optimal adhesion at intermediate protein density, proving that more is not always better.

## Introduction

Complex living organisms are defined by hierarchical assemblies of different types of tissues. At the point where these structures are connected, as well as at their peripheries, interface is created. These can be either sharp, like an epithelium, or more gradual, like the ligand–bone interface in the knee joint. The transition from bone to ligament is marked by a decreasing mineral content gradient on a collagen fiber scaffold (1). As a result, a stiffness gradient exists along the interface. In line with the decrease of stiffness, the four regions are respectively occupied by osteoblasts, hypertrophic chondrocytes, chondrocytes, and fibroblasts, forming a cellular gradient which defines the hierarchical organization of the tissue. Another process directed by gradients is angiogenesis in wound healing, where endothelial cells migrate to the perivascular area and then grow into new vessels under the direction of nutrients and cytokine concentration gradients (2). In addition to stiffness gradients and gradients in the concentration of signaling molecules, also gradients in surface-attached molecules guide cellular behavior in living organisms (3). In consequence, there is a need to investigate how cell behavior such as adhesion and migration is influenced by gradients. Besides replicating gradients found in tissues, continuous gradients of surface-attached molecules also represent an attractive means for the high-throughput screening of the effect of ligand concentration on cells (4). Since in vitro studies with cells are predominantly carried out in (quasi) planar systems using either completely flat (2D) or microstructured (2.5D) surfaces, the influence of gradients on cellular behavior is also primarily studied in that geometry.

Fabrication strategies for surface gradients include light illumination (5), microcontact printing (6), and diffusion (7). However, as of today, most of the resulting gradient materials either lack precise control over the molecular arrangement at the nanoscale or have potential toxicity to cells (8–10).

Recently, a dip-coating approach for varying the density of RGD on a solid substrate by changing the dipping velocity has been demonstrated. This approach is highly capable of producing a surface density gradient of the deposited molecules (11). Nonetheless, it does not afford precise control over the organization and orientation of the deposited molecules. Additionally, predicting the areal density of molecules deposited from a bulk solution during dip coating typically is not straightforward. Therefore, here, we use the Langmuir–Blodgett (LB) technique to fabricate protein-based ultrathin 2.5 D films with a protein density gradient. By assembling the molecules in a monolayer prior to deposition, we gain precise control over their orientation, while adjusting their areal density via compression. The LB transfer is a vertical lifting deposition similar to dip coating, where objects that are assembled at the air–water interface are deposited on a solid substrate under the action of convection, intermolecular interactions, and capillary force (12). To create a gradient, a protein layer at the air–water interface is compressed at a rate of $v_{C}$ and at the same time the substrate is lifted with a speed $v_{L}$. When the value of $\;v_{C}$ is set greater than the rate at which material is deposited on the substrate, with the decrease of surface area, the molecules will be arranged in a more compact way. Therefore, assuming a constant lifting speed $v_{L}$, the slope of the concentration gradient increases with $v_{C}$. While a theoretical calculation of the gradient length ($L_{G}$) and slope are straightforward, one has to be aware that the deposition process can be influenced by further factors such as capillary forces, evaporation, and the movement of the three-phase contact line between substrate, subphase, and air (13), which can e.g. lead to striping patterns due contact line pinning (14). Avoiding those effects requires optimization of experimental parameters for the chosen system, e.g. substrate orientation, tilt, and hydrophilicity.

**Fig. 1.**
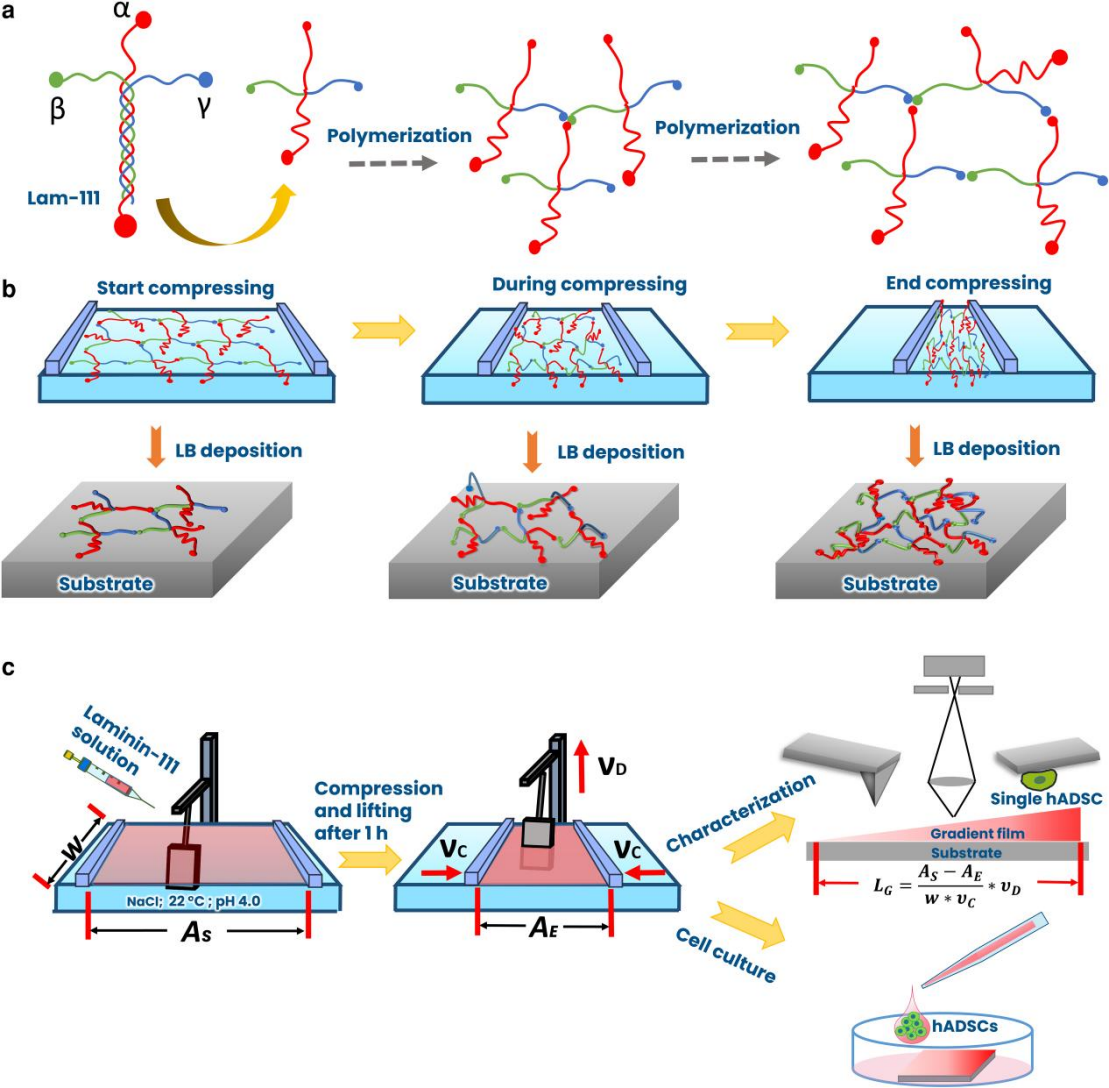
Schemes of a) the structure of Lam-111 and polymerization. The representation of the long arm consisting of a triple-helix is simplified for clarity, b) the proposed organization of Lam-111 at the air–water interface during compression together with the resulting thickness profiles of the deposited film, and c) fabrication and characterization of gradient Lam-111 LB-film via microscopy and by culturing hADSC on the film. *w*, the width of the barrier; *A_S_*, start area of the surface; *A_E_*, end area of the surface; *ν_c_*, compression speed; *ν_D_*, lifting speed of the substrate; *L_G_*, gradient length.

**Fig. 2.**
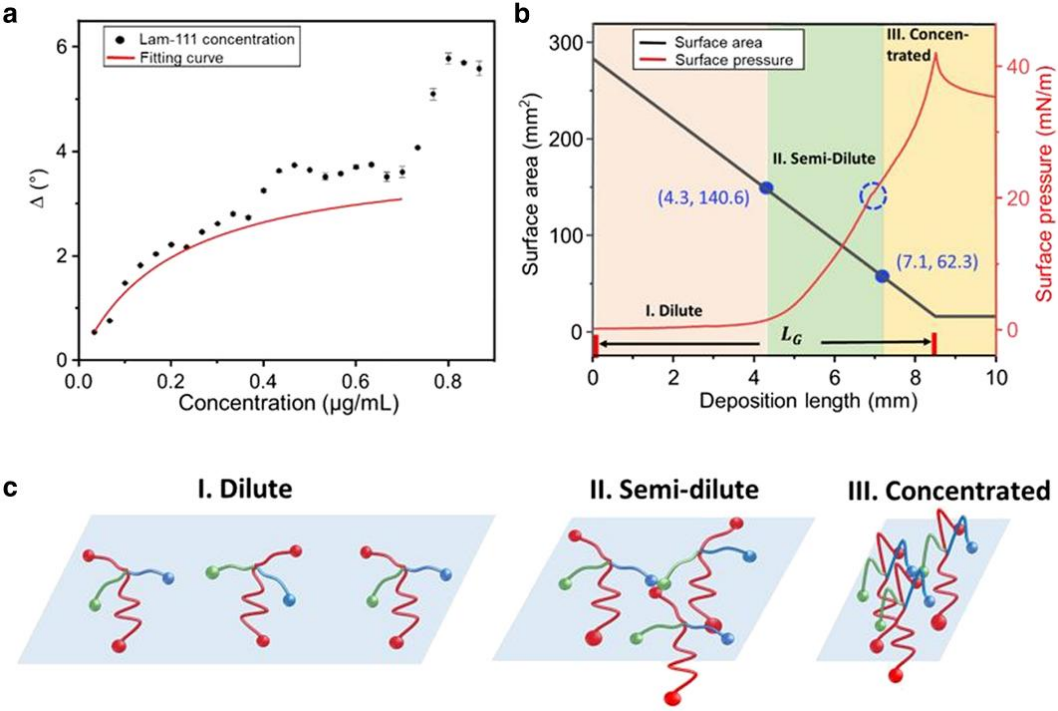
a) Type IV adsorption isotherm of Lam-111 at the air–water interface in the plot of ellipsometric angle Δ vs. Lam-111 bulk concentration. The red line shows the fitting curve of the first step adsorption from 0 to 0.7 µg/mL. b) Behavior of Lam-111 monolayer during the deposition via lifting the substrate from the subphase. The red curve stands for surface pressure. The black curve stands for the trough area during compression. The x-axis represents the length of the substrate lifted above the liquid surface. The blue dashed circle on the compression isotherm indicates the kink at 20 mN/m. The points (*d* = 4.3 mm, *A* = 140.6 cm^2^) and (*d* = 7.1 mm, *A* = 62.3 cm^2^) on the trough area curve divide the layer's state into three phases: dilute (*d* = 0–4.3 mm), semi-dilute (*d* = 4.3–7.1 mm), and concentrated (*d* = 7.1–10.0 mm) phase. (c) Schematic of the hypothesized organization of Lam-111 molecules in three phases.

For this work, we chose laminin-111 (Lam-111), which is a natural protein in the extracellular matrix (ECM) (15), endowing multibinding sites for integrin and chemokines (16). Moreover, it is known to form ultrathin polymerized structures with its α, β, and γ N-terminals (17), and we hypothesize that the presence of a cohesive 2D network structure can help to suppress the aforementioned pattern formation (see Fig. 1a). The prevalent usage of Lam-111 in cell research also makes it an ideal protein for investigating how such concentration gradients affect cellular responses such as adhesion. By means of ellipsometry (Fig. 2a), we developed a hypothesis for the state of the Lam-111 film at the air–water interface, which is illustrated in Fig. 1b. At the chosen experimental conditions, which refer mainly to the amount of Lam-111 solution applied to the interface, the molecules form an expanded network at the beginning of compression and a monomolecular film is transferred onto the substrate at this point. When the film is compressed to a smaller area, the extended peptide chains are deformed and start to buckle, causing a moderate increase in the film thickness. Upon further compression, the molecules reach the maximum possible packing density of a true monolayer, and compression beyond that point forces them to pile up, leading to a further increase in the thickness of the film. Based on this model, the experiments are designed as shown in Fig. 1c. After self-assembly of Lam-111 in the rather dilute state the substrate, which is previously immersed in the subphase, is raised while the barriers are compressing the monolayer. In that way, the temporal evolution of all packing states of the molecules in the layer is recorded spatially along the deposition direction (18). To understand how such a straightforward approach can regulate protein density and cell response, we carried out a comprehensive characterization of the gradient film. Most of the analysis was performed using different atomic force microscopy (AFM) techniques rendering film thickness, Young's modulus, and the adhesion force of a single cell along the gradient. The wettability along the gradient was recorded by a sessile drop device. The protein surface density was measured by confocal microscopy through immunostaining of laminin.

Furthermore, human adipose-derived stem cells (hADSCs) were seeded onto the gradient films to investigate the influence of laminin surface density on cell attachment, thereby shedding light on the underlying mechanisms governing cellular responses.

## Results and discussion

### Quantifying laminin adsorption via ellipsometry

To quantify the laminin molecules adsorbed at the air–water interface, null ellipsometry was used. According to previous work in our lab, the protein surface excess Γ (adsorbed amount of substance per unit area of surface) of an ultrathin protein layer (thickness < 20 nm) is proportional to the ellipsometric angle Δ:


(1) Δ=β*Γ

and


(2) $$\Delta =\Delta_{p}-\Delta_{s},$$

where *β* is a constant coefficient, $\Delta_{p}$ and $\Delta_{s}$ are the ellipsometric angle of protein layer and clean subphase, the latter being 180° in an ideal case (19).

The plot of the ellipsometric angle *Δ* against Lam-111 concentration in Fig. 2a illustrates that the adsorption behavior of Lam-111 follows a two-step (type IV) adsorption isotherm. The first step occurs within the concentration range of 0–0.7 µg/mL, and the second step is observed between 0.7–1.0 µg/mL. In the initial step, Δ increases along the increasing total protein concentration and then reaches a plateau at around 4°. The saturation observation at high concentration is likely due to the surface being completely occupied with a layer of adsorbed molecules. This part of the curve can be described with the Langmuir adsorption isotherm of a monolayer using the following equations:


(3) $$\;\theta =\frac{K\;*\;c}{1+K\;*\;c}.$$

With


(4) $$\theta =\frac{\Gamma }{N},$$

where *K* is the Langmuir adsorption equilibrium constant, *c* is the bulk protein concentration, and *N* is the total number of adsorption sites, which can be approximated as the ratio between trough area and minimal molecular area (20).

Therefore, $\frac{K\;*\;c}{1+K\;*\;c}=\frac{\Delta }{\beta \;*\;N}$. Since both *β* and N are constant, we define a constant A=β*N. Thus, the function


(5) Δ=A*K*c/(1+K*c)

is used to fit the Lam-111 adsorption in the first step, resulting in *K* is 4.9 mL/µg. The complete fitting information can be obtained in Table S1. For the fabrication of the gradient film, 10 µg Lam-111 was spread onto 50 mL of subphase. According to the former adsorption experiment, with a concentration of *c* = 0.2 µg/mL, the calculated θ predicts that 49.5% of the trough surface was covered with Lam-111 molecules upon reaching the adsorption equilibrium. The ellipsometric adsorption isotherm shows that when the bulk concentration is increased above 0.7 µg/mL, the molecules overlap and start to form multilayers. In that sense, increasing the bulk concentration has a similar effect on the monolayer as compression.

### Compression and transfer

The fabrication of the gradient film was carried out on a Langmuir trough, by compressing the protein layer at the air–water interface to a target area. At the same time, the film was transferred into a raising Si wafer with a size of 10 mm × 10 mm. Since the substrate was tilted with an angle of 30° to the vertical dipping controller, the deposition speed ($\upsilon_{D}$) was greater than the lifting speed (1 mm/min): ($\upsilon_{D}$) = 1 mm/(min × cos(30°)) = 1.15 mm/min. The gradient length $L_{G}\;$ can be calculated in the equation below:


(6) $$L_{G}=\frac{A_{S}-A_{E}}{w\;*\;\upsilon_{C}}\;*\;\upsilon_{D},$$

where $A_{S}$ stands for the start area of the trough, mm; $\;A_{E}$ stands for the end area of the trough, w stands for the width of the trough, and $\;\upsilon_{C}$ stands for the compression speed. With $\upsilon_{C}$ = 46.75 mm/min and w = 80 mm, the calculated gradient length is 8.24 mm. The organization of the Lam-111 monolayer during the fabrication of the gradient LB-film transfer can be deduced from the surface pressure (*π*) vs. gradient length (*d*) isotherm. It is plotted in Fig. 2b together with the trough area (*A*). Two critical points (*d* = 4.3 mN/m, *A* = 140.6 cm^2^) and (*d* = 7.1 mm, *A* = 62.3 cm^2^) are marked on this curve. At the first point, the trough surface was reduced to half of the starting area. Based on the results shown in Fig. 2a, one expects the surface to be completely covered with laminin from this point onwards. At the second point, a subtle bulge is observed at *π* = 20 mN/m on the compression isotherm curve. Such discontinuities usually indicate a transition in the arrangement of the molecules. In line with the established theories for macromolecules in 2D, we suggest that during the fabrication process, the adsorbed Lam-111 molecules pass through three phases: dilute, semi-dilute, and concentrated phase. The expected structures of these phases are depicted in Fig. 2c. The dilute phase is present from deposition length *d* = 0 to 4.3 mm. In this phase, the surface pressure is close to 0 mN/m, meaning that there are no repulsive interactions between the spread laminin molecules. These molecules exist as individual entities and on average each one occupies an area on the surface that is larger than its own size. Therefore, the local protein concentration is highly discontinuous and anisotropic. The semi-dilute phase starts at the length of *d* = 4.3 mm, where the surface area of the trough is reduced to half its initial value, indicating that the film's surface is completely covered with a layer of adsorbed Lam-111 molecules. Here, the molecules come into contact and subsequent compression of the film results in deformation of the molecules and an almost linear increase in surface pressure. Once further compression of the molecules becomes energetically very demanding they start to overlap in the concentrated phase. As laminin molecules are water soluble, one would expect the molecules to desorb into the bulk solution in this stage. However, due to the polymerization of Lam-111, an insoluble layer is formed at the air–water interface (21), which resists further compression instead of desorbing to the bulk subphase. All in all, the temporal evolution of the film through the three phases is recorded on the moving substrate where a film with a gradient in protein density and thickness is deposited.

### Wettability of Lam-111 gradient LB-film

The variation of molecular packing density is expected to affect its wettability.

**Fig. 3.**
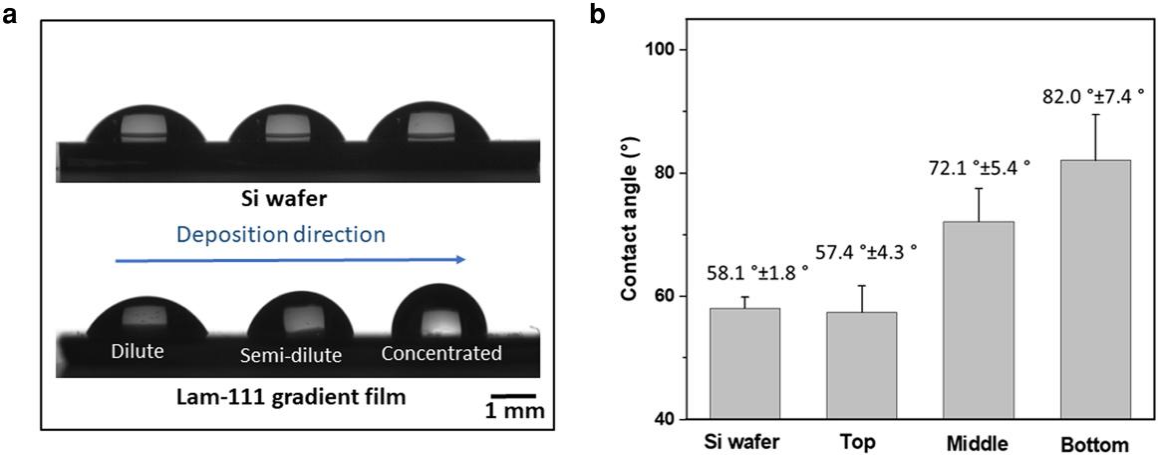
Water contact angle measurements of Si wafer and Lam-111 gradient film. a) Images of three droplets on bare Si wafer and Lam-111 gradient film. b) Results of the water contact angle measurements.

Referring to the optical overview of the whole gradient film taken by digital spectroscopy (see Fig. S1), the film should have at least three variations at its dilute, semi-dilute and concentrated parts. Fig. 3a shows the optical profiles of water drops on a Si wafer and a Lam-111 gradient film at the dilute, semi-dilute, and concentrated zones of the gradient (starting from the dilute zone). The three droplets present similar shapes on the Si wafer, while those on the gradient film are markedly different. The derived contact angles are displayed in Fig. 3b. Although the contact angle of the Si wafer (58.1°±1.8°) and the dilute part (57.4°±4.3°) of the gradient film are similar, that of the semi-dilute and concentrated parts increased to 71.2°±5.4° and 82.0°±7.4°, confirming the presence of a gradient material. A plausible explanation of the increased water contact angle is that Lam-111 molecules self-assemble at the air–water interface with the hydrophobic groups exposed in the air and the hydrophilic groups towards the water. During the LB transfer, the protein monolayer transitioned from the dilute phase to the concentrated phase. Therefore, the areal protein density increases so does the density of hydrophobic groups exposed to the air. Particularly under conditions of high compression, the hydration state of the molecules can change due to the squeezing out of water between the hydrophilic groups of the protein molecules. Consequently, this results in a tendency of reduced affinity to water along the deposition direction.

### Morphology of Lam-111 gradient LB-film

**Fig. 4.**
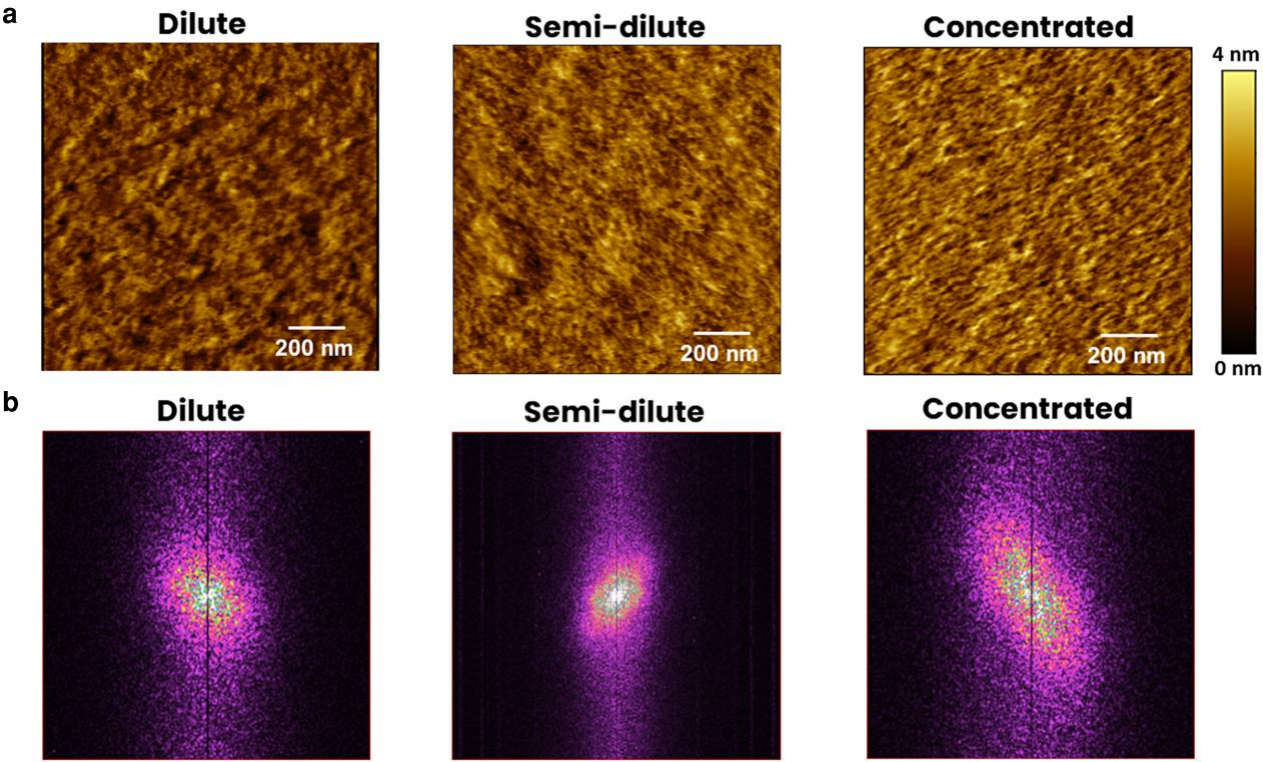
a) AFM images at the dilute, semi-dilute, and concentrated parts of Lam-111 LB-film along the lifting direction in PBS buffer at 22°C. b) 2D-FFT patterns of the AFM images of the dilute, semi-dilute, and concentrated parts. The vertical lines in the middle of the 2D-FFT images are caused by the AFM scanning direction.

To analyze the morphology of protein-based scaffolds in biological environment, the Lam-111 gradient film was characterized using AFM under contact mode in Phosphate-Buffered Saline (PBS) at 22°C. The nanostructure of the dilute, semi-dilute, and concentrated parts of the gradient film are illustrated in Fig. 4a. The dilute part of the film presents a low protein density film consisting of many dark and bright areas, which reflects the dilute phase of molecular arrangement, presence of a structure with locally fluctuating protein density. While we suggest that the molecules are not tightly packed, the blank substrate between the molecules cannot be resolved due to the lateral resolution limit of the AFM tip (typically ∼10 nm). In the semi-dilute part of the gradient film, there exists a denser arrangement of the protein molecules and many fiber-like structures, matching the above assumption of semi-dilute phase in the central zone of the gradient. These fiber-like structures become highly concentrated and show a recognizable orientation in the concentrated part of the gradient. The fiber-like structures are probably formed under the strain generated by compression of the laminin network (21). Alternatively, the vertical deposition could have rearranged the network strands by stretching the film through the capillary force. Altogether, the three representative morphologies are consistent with the increasing areal protein density and the phases described above. To confirm the orientation and appearance of periodic feature formation through a statistic method, a 2D-fast Fourier transform (FFT) of the three AFM images was carried out in Fig. 4b. For the dilute part, only a broad maximum at the origin is observed, meaning the protein molecules are randomly distributed. The semi-dilute part depicts that the frequency distribution is narrower and a weaker orientation appears, implying that the structures were deposited on the substrate with a certain orientation and similar sizes. At the concentrated part of the gradient, the center of the FFT is a broader ellipse with a diagonal orientation and with frequency centers. Combined with the morphology presented in the AFM image, it can be concluded that these fiber-like aggregations indeed have an alignment and a somewhat regular distance. The average roughness also increases along the gradient, from about 1.4 nm to 2.8 nm when measured on 10 µm × 10 µm scans (see Fig. S2). While nanoscale roughness can influence cellular behavior (22, 23), the variation of the roughness at a distinct gradient position is of a similar magnitude as the variation along the gradient. That means that any roughness effects on the interaction with cells are most likely obscured by averaging several measurements taken at the same gradient length.

### Thickness of Lam-111 gradient LB-film

**Fig. 5.**
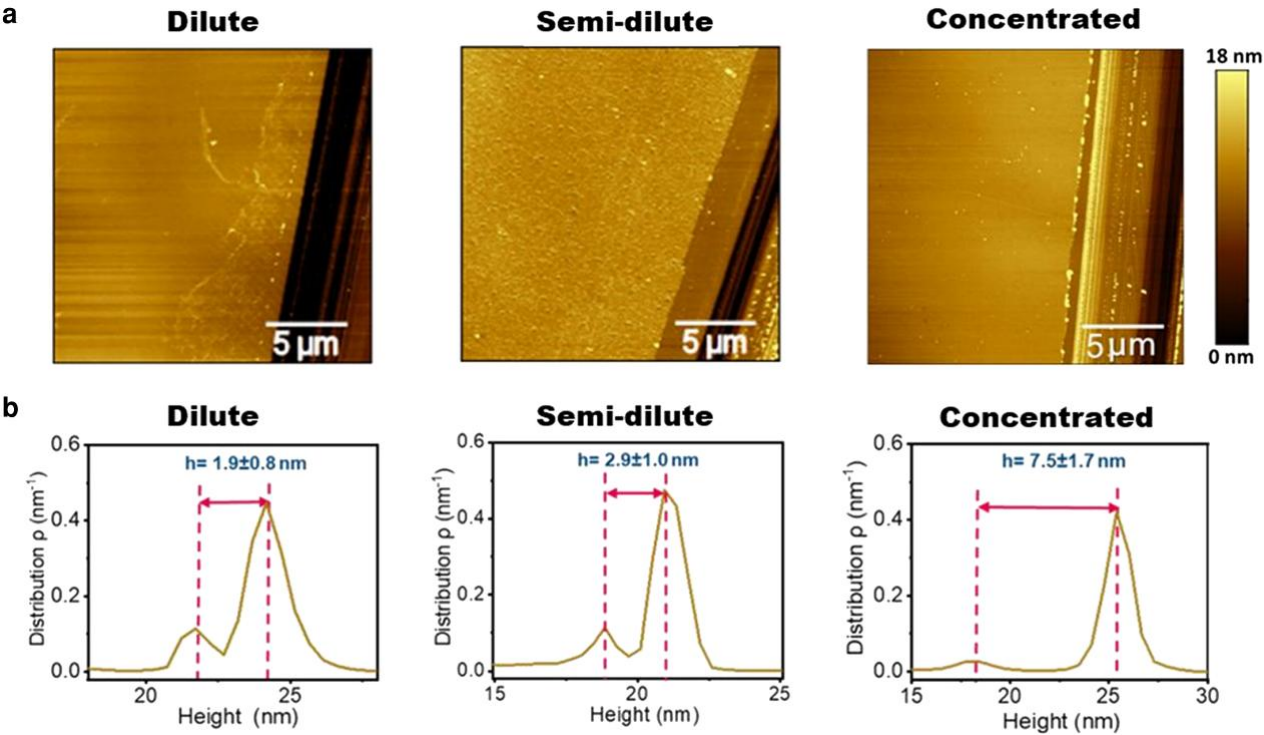
a) AFM images of Lam-111 LB-film with scratches at the dilute, semi-dilute, and concentrated parts of the gradient in PBS buffer at 22°C. b) Height distributions of corresponding AFM images and the calculation of the thickness.

Fig. 5a shows the AFM images of scratches made at the dilute, semi-dilute, and concentrated parts of the film and the corresponding height distributions of these images are presented in Fig. 5b. The results show that the film thickness at the selected points at the dilute, semi-dilute and concentrated parts of the gradient are 1.9 ± 0.8 nm, 2.9 ± 1.0 nm, and 7.5 ± 1.7 nm, respectively. According to previous studies, the long arm of Lam-111 is around 77 nm long while the other three short arms have a length of about 34 nm (24). These values are much larger than the thickness we obtained. This demonstrates that on the substrate, the laminin molecules are lying flat. The molecules are highly flexible and can easily collapse upon drying of the film, so the orientation before transfer might have been different. In a recent investigation with Lam-111, we found that the self-assembled monolayers have a thickness of about 3 nm (25). The lower thickness found at the dilute part of the film is in agreement with the hypothesis that the molecules are not tightly packed at the beginning of the transfer. The film formed in the semi-dilute regime has the same thickness as the self-assembled monolayers. At the concentrated part of the film, the molecules are either overlapping or forced into a more 3D conformation, resulting in a thickness more than double that in the semi-dilute state.

### Stiffness of Lam-111 gradient LB-film

**Fig. 6.**
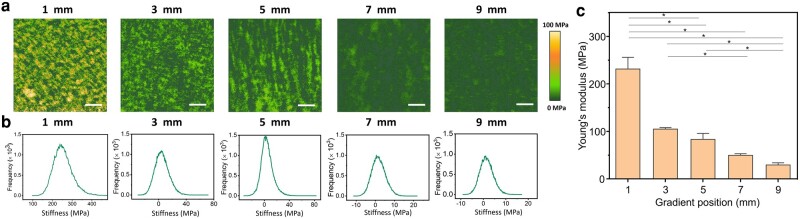
a) Stiffness maps of measured sites at 1, 3, 5, 7, and 9 mm of the gradient Lam-111 film. All scale bars are 200 nm. b) The corresponding histograms of stiffness maps. c) Bar chart summarizing the average Young's modulus values of corresponding measured points. Three points were averaged for each position. Error bars are standard error of mean, and significant differences (one-way ANOVA with Bonferroni test, *n* = 5, *P<<0.05).

Since the cytoskeleton of cells can be modulated by the adhesion between actin and the surrounding ECM proteins, stiffness is a key mechanical parameter of material surfaces when it comes to controlling cellular interactions. The established method to evaluate the stiffness of the substrate experienced by the cells is conventional tensile testing; however, this method may not accurately capture the dynamic interactions experienced by adherent cells. Here, we use AFM in quantitative imaging (QI) mode in the liquid environment to characterize the mechanical properties of the soft gradient biomaterials at the nanoscale. Stiffness maps of points measured at the 1, 3, 5, 7, and 9 mm of the gradient Lam-111 film are shown in Fig. 6a. The averaged Young's moduli extracted from the stiffness maps are shown in Fig. 6b. Compared to the stiffness of the bare Si wafer, which is about 7 GPa (see Fig. S3), the stiffness of the ultrathin layer of laminin is on the order of MPa, dramatically altering the properties of the rigid substrate. In fact, the stiffness continuously reduced from 231 ± 31 MPa at 1 mm to 29 ± 6 MPa at 9 mm. As the stiffness maps do not show any pixels with stiffnesses on the GPa level, we infer that the AFM tip is not directly tapping onto the silicon substrate, and therefore the progressive shift in average stiffness does not arise from a mixing of hard substrate and soft proteins. Rather, the tip is probing different deformation modes of the predominantly α-helical proteins. When the thickness is below the monolayer level, the molecules are lying flat on the substrate. Indentation testing will therefore mainly probe the radial compressibility of the α-helices. For collagen fibrils, the stiffness in that deformation mode was found to be in the GPa range in air (26) and in the 1–170 MPa range in water (27, 28). The broad range arises from the sensitivity to numerous factors, including measurement method, collagen source and mechanical analysis. As the thickness of the layer increases, so does the fraction of molecules and molecular segments that are not adsorbed to the surface. These segments can be deformed more easily by sliding, bending and shearing, thereby contributing to the decreased Young's modulus as the layer thickness increases.

### Protein density of Lam-111 gradient LB-film

**Fig. 7.**
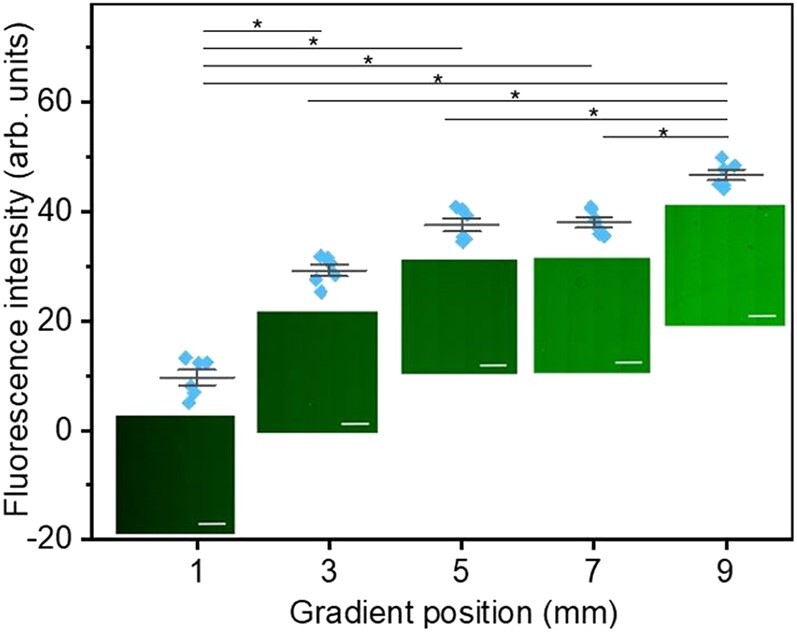
Mean Fluorescence intensity per pixel of six randomly selected zones with size 0.3 mm × 0.3 mm at the 1st, 3rd, 5th, 7th, and 9th mm of the gradient film, individually. The significance of differences was determined by one-way ANOVA with Bonferroni test, *n* = 6, *P<<0.05). Images of NHS-fluorescein labeled laminin at corresponding points were taken by confocal microscopy. All scale bars are 200 µm.

To determine the areal density of Lam-111, the protein was labeled with NHS-fluorescein the sample was scanned with confocal fluorescence microscopy. Compared to immunostaining with antibodies, this one-step staining is faster and requires fewer steps which minimizes the damage to the ultrathin film. The fluorescence intensity of 6 randomly selected zones with a size of 0.3 mm × 0.3 mm at the 1st, 3rd, 5th, 7th, and 9th mm of the gradient film were processed via Image J. Representative images at the corresponding positions are shown in Fig. 7 together with the mean fluorescence intensity per pixel, with the blank wafer subtracted as background. As expected, the color of the images increased from dark green to light green, confirming the presence of a protein density gradient.

### Cell adhesion force performed on Lam-111 gradient LB-film

To investigate the mechanotransduction of the integrin-mediated cell–ECM adhesion in the microenvironment with multibiophysical cues, the interaction between cells and Lam-111 gradient LB-film was quantified using AFM-based cell adhesion force spectroscopy (29) with a single hADSC as the scanning probe.

**Fig. 8.**
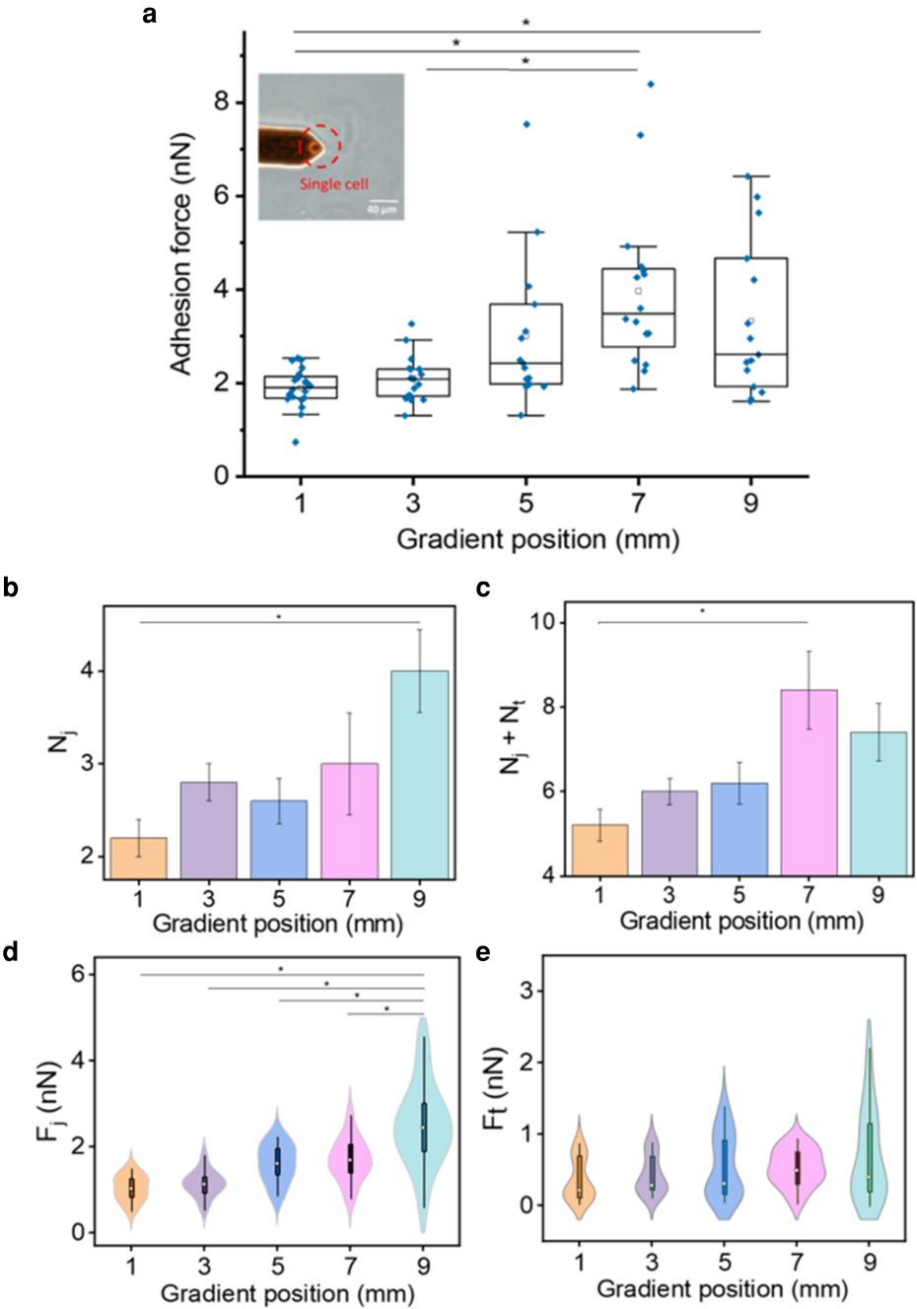
a) The adhesion force between the film and a single hADSC captured by a tipless AFM cantilever measured at the 1, 3, 5, 7, and 9 mm of the gradient film. The top and bottom of the box stand for the 75th and 25th percentile, individually. The middle line in the box stands for the median while the white cubic stands for the mean value. The upper and lower whiskers indicate the maximum and the minimum. The diamonds above boxes are outliers. The significant differences were determined by one-way ANOVA with Bonferroni test, *n* = 15, *P<<0.05. Inset: Optical microscopy image of a tipless cantilever with a single hADSC attached. b) The average number of jumps Nj at the gradient positions. c) The total number of unbinding events Nj + Nt at the gradient positions. d) The force of jumps Fj at the gradient positions. e) The force of tethers Ft at the gradient position. Fj and Ft are transferred into positive value. Error bars are the standard error of mean. The significance is labeled according to one-way ANOVA analyzed results, Bonferroni test, *P<<0.05. d) and e) are combination of density plot and box plot and the white dot stands for the median value.

In Fig. 8a, an optical photo of a spherical cell with normal size on the cantilever confirmed the viability of the probed cells. And the statistical analysis of the maximum adhesion force along the gradient acquired from the retract force–distance (F–D) curve was plotted in the form of box chart. The mean values at corresponding length were listed in Table S2. The adhesion force along the gradient gradually increased from 1.9 ± 0.1 nN at 1 mm to 4.0 ± 0.4 nN at 7 mm and decreased to 3.3 nN at 9 mm. We deduce that the tendency of the cell adhesion force change resembles the Lam-111 compression isotherm. It shows a modest increase of 0.2 nN between the measured points at 1 and 3 mm. Afterward, it increases more rapidly from 0.9 nN from 3 to 5 mm, in which region the surface pressure also rises steeply. The strongest adhesion force is observed at 7 mm, coinciding with the formation of a compact protein monolayer. In principle, one could expect that the adhesion force increases further beyond that point, as a higher concentration of laminin molecules at the attachment site would lead to a greater cell-film interaction via integrin binding. A plausible explanation for the fact that the adhesion force decreases at 9 mm could be the distorted conformation of the laminin molecules in this section of the gradient (see Fig. 2c). Intense compression of the Laminin network prompts the aggregation and overlapping of polymerized chains, as suggested by AFM (Fig. 4c). Consequently, a share of the binding sites of Lam-111 is likely obstructed or buried below the surface. Whether or not cells can unravel the laminin film to expose these binding sites will depend on the force that is being generated by the cells and the time given for them to do so. Cells are exerting traction tensions on the order of 5 kPa (30), with individual focal adhesions (FAs) developing traction forces on the 1–30 nN level (31). If such traction forces act on individual nanometric fibrils, they generate tensions in the GPa range, which would be sufficient to overcome the modulus of the fibrils and trigger a reorganization and accumulation of the ECM around the FAs. This can be observed quite clearly around the hADSCs cultured on the gradient films (see Fig. S6). On the other hand, the cell–material contact time in the adhesion force measurement is only 10 s, and the force with which the cells are pressed into the film is 5 nN, which results in a kPa level contact pressure given the area of the cells is in the 10–100 µm^2^ range. That means the FAs would remain at the surface of the films with MPa stiffness so that the cells will also not be able to feel the underlying substrate directly.

In a typical retract F–D curve, the maximum detachment force is comprised of jump events and tether events, both of which represent unbinding events resulting from the stretching of adhesion complexes between the cells and the surface (29). The jump events are characterized by a “sawtooth” shape and signify the disruption of individual integrin adhesion complexes from the substrate. Meanwhile, tether events manifest as step-like changes with plateaus and occur during the extraction of the cell membrane nanotube (32). The average number and force of these unbinding events were calculated by the JPK Data processing software. In Fig. 8b, there is a generally increasing tendency in the number of adhesion units from 2 to 4 along the gradient. The high value at 3 mm might be because the cells were more active at the onset of the experiments. As depicted in Fig. 8c, the total number of unbinding events Nj + Nt exhibits a progressive increase from the 1 to 7 mm along the gradient. This trend aligns with the observed variation in adhesion force, which peaks at the 7 mm mark and slightly decreases thereafter. Corresponding to the Nj plot, the jump force Fj also demonstrates an overall ascent along the gradient, ranging from 1.03 nN to 2.50 nN. Conversely, the tether force Ft tends to be smaller than Fj on average, as the tether events precede the jump events, and at that time the adhesion force returns to baseline upon cantilever retraction. The highest value of Ft is only 0.49 nN at 7 mm and decreases at 9 mm. The results of this experiment indicate that both jumps and tethers contribute to the maximum adhesion force. Numerous studies in the field of cell–ECM interaction and biomaterial adhesion have concluded that the number of binding sites for cells increases with protein density. According to the fluorescence staining experiment mentioned above, the Lam-111 density of the gradient film is expected to peak at the end, while the cell adhesive force decreases at 9 mm. This observation appears to contradict the aforementioned conclusions. Nevertheless, it is well-known cells tend to generate larger FA forces on rigid substrates compared to soft substrates. Given that the Young's modulus decreased with laminin density, the reciprocal interplay between elasticity and laminin density can generate a maximum of cell adhesion force at less than maximal protein density. The same experiment was carried out using HaCaT cells as probes. Here, the adhesion force along the gradient shows only minor variations, narrowly ranging from 1.9 nN to 1.5 nN (see Fig. S4). Instead of increasing, the adhesion force decreases along the gradient from 1 to 5 mm, and subsequently stabilizes at 1.5 nN (see Table S3). The number of jumps Nj, the total unbinding events Nj + Nt, the force of jumps Fj and the force of tethers Ft all vary in a narrow range without clear tendency, except for the total number of unbinding events, which showing a decreased tendency along the gradient. Since this result matches the maximum adhesion force, a possible suggestion is that in HaCaT cells the tethers contributed more to the adhesive force than the tethers in the hADSCs. This phenomenon does not align with the observations using hADSCs. The differing performance of cell adhesion on the gradient Lam-111 film between hADSCs and HaCaT cells can be explained by their distinct binding effects of integrins on certain ECM proteins (33).

### Cell distribution on LB Lam-111 gradient films

To verify that these biophysical gradients influence cell behavior, the distribution of hADSCs on the gradient sample was evaluated through immunostaining 24 h after cell seeding. Before this experiments, a stability test of Lam-111 film during the cell culture since cells can digest, reconstruct and secret laminin (34). The test of the specifical immunostaining of the coated mouse Lam-111 showed that the laminin film can maintain the gradient structure after 24 h culture (see Fig. S5).

**Fig. 9.**
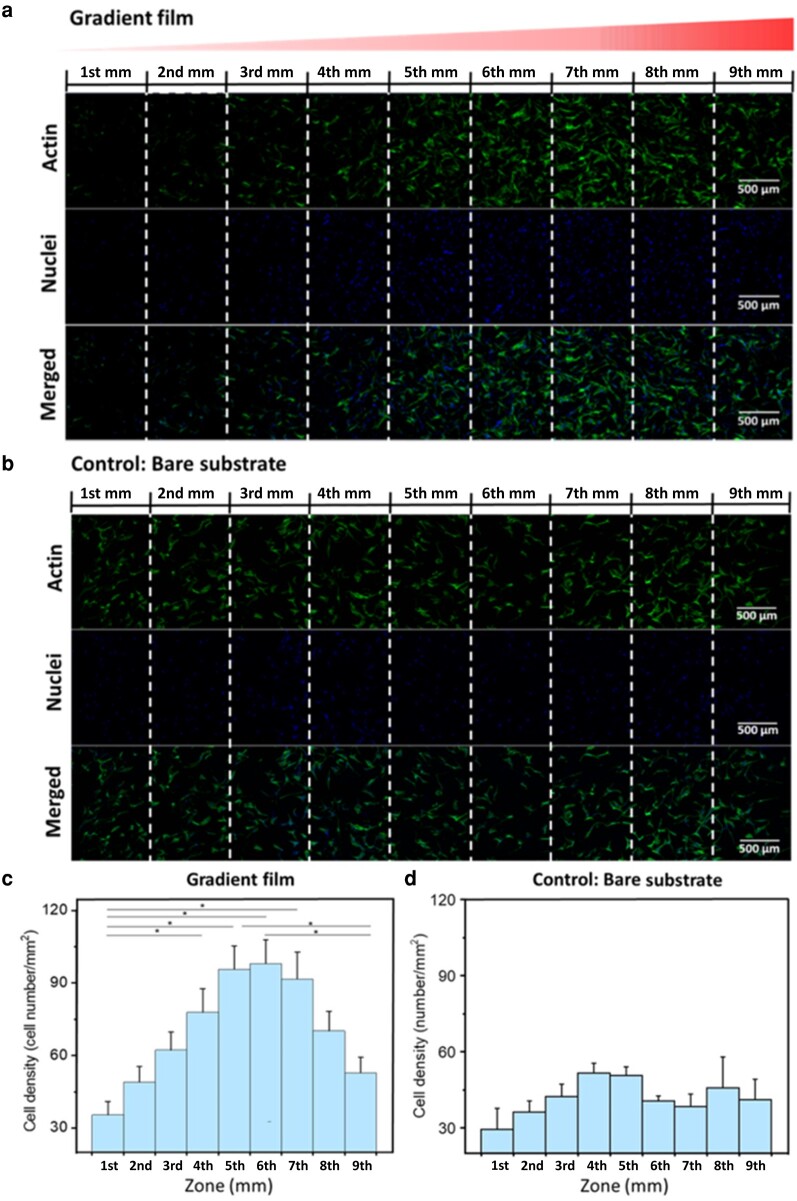
a) and b) are confocal images of hADSCs cell distribution on the gradient Lam-111 film and on bare fused Si wafer. The cells were fixed and stained after 24 h of culture at 37°C, 5 (v/v) % CO_2_ with a seeding density of 1.0 × 10^4^ cell/cm^2^. Green stands for the actin and blue stands for the nuclei. The scale bar is 500 µm. c) and d) are statistical analyses of the distribution of hADSCs on gradient Lam-111 film and bare substrate. Cell density was calculated by homogeneously dividing the films into 1 mm × 8 mm slices. Error bars are the standard error of mean, and significant differences (one-way ANOVA with Bonferroni test, *n* = 9, *P<<0.05).

A complete substrate analysis was performed using confocal microscopy, involving dual-channel imaging with multilayers. Fig. 9a shows the gradient's entire length (9 mm × 1.2 mm), evenly divided into nine zones. The cell density increases towards the 7th mm and then noticeably attenuates towards the gradient's terminus. The fluorescence image of the control substrate (bare Silica), recorded and processed in the same way, is illustrated in Fig. 9b. The cells show no obvious preference for any position on the substrate. The statistical evaluation is depicted in Fig. 9c. Evidently, the cell count per square millimeter increases along the gradient, peaking at the 6th zone with a threefold increase from the initial value. Subsequently, it gradually declines towards the end. Noteworthy, the cells are clustered within the 5th mm, 6th mm, and 7th mm, each exhibiting relatively uniform cell densities. The statistical analysis of the control samples (Fig. 9d) illustrates that the cells do not show a preferential tendency and also exhibit less attachment. The distribution of stem cells broadly aligns with cell adhesion force measurements, indicating that regions with heightened cell adhesion forces either attract or retain more cells—or both. It is plausible that the 24 h interval between cell seeding and fixation allowed ample time for cells to migrate, particularly for cells initially adhering in areas characterized by low protein density.

The distribution of HaCaT cells on Lam-111 gradient films was investigated analogously (see Fig. S6). The immunostaining images of HaCaT cells on a gradient substrate and a bare substrate are illustrated in Fig. S6a and b. Compared to hADSC, more HaCaT cells were observed on both gradient and bare substrate. Additionally, more cell colonies were formed on the gradient substrate than on the bare substrate. The quantitative analysis of the cell density reveals that there is a slight increase in cell number from 1st mm to 7th mm zone followed by a pronounced decrease (Fig. 6c and d). Unexpectedly, HaCaT cells have a larger density on bare Silica. Also, there is no direct correlation between the distribution of HaCaT cells on the gradient film and their adhesive force. Fluorescence labeling the gradient film around the HaCaT cells shows that the cells are reorganizing the film as well, leading to an increase in laminin density at the outline of the cells, together with depletion zones where the laminin was withdrawn towards the cells (see Fig. S5). However, this rearrangement is very localized, suggesting that the cell did not migrate over large distances, in line with the homogeneous cell distribution. Above all, it can be concluded that hADSC responds much stronger to the Lam-111 gradient than HaCaT cells.

### Quantification of FA on LB Lam-111 gradient films

For a better understanding of the intergrin-mediated FA of the cells on this gradient substrate, the quantification of vinculin expression, an essential FA protein for mechanosensing (35), was applied to the cells cultured for 12 h. Then the confocal fluorescence microscopy images of vinculin were analyzed with particle measurement using image J.

**Fig. 10.**
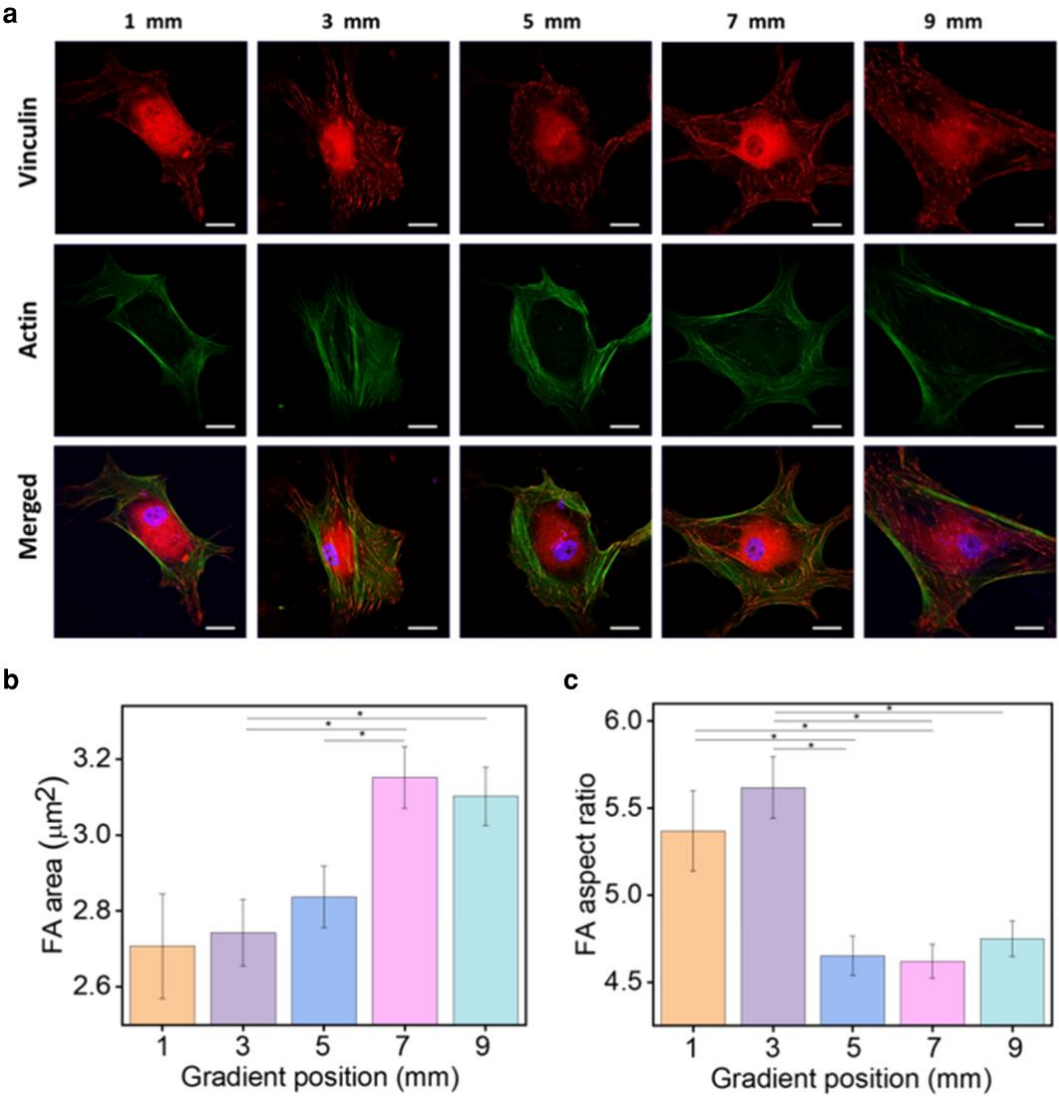
a) The immunostaining images of the vinculin (red) expression and the corresponding images merged with Phalloidin (green) and nuclei (blue) of hADSCs cultured on the gradient Lam-111 LB-film for 12 h. Scale bar is 20 µm. b) and c) are the statistical analyses of mean focal adhesion area (FA) area and mean focal adhesion (FA) aspect ratio. At each position, eight images of individual cells were processed. Error bars are the standard error of mean, and significant differences (one-way ANOVA with Bonferroni test, *P << 0.05).

In Fig. 10a, individual stem cells at 1, 3, 5, 7, and 9 mm of the gradient are depicted, labeled with anti-vinculin antibody (red), and corresponding images merged with actin (green), and nuclei (blue). Vinculin appears as rod-like lines predominantly located at the ends of actin fibers. Additionally, it was observed that cells were more polarized at 1 and 9 mm of the gradient compared to those in the middle zone, particularly at 7 mm where cells spread outward and formed many protrusions. To statistically analyze the size of vinculin along the gradient, eight images of individual cells at the five positions were processed using the particle measurement function in Image J. The mean area and aspect ratio of rod-like vinculin were calculated and presented as bar plots. As Fig. 10b presents, the FA area exhibited a stepwise increase within the gradient range of 1–7 mm, followed by a decrease thereafter. However, based on the findings reported by Benjamin and Alexander's group, the FA area on rigid substrates exhibits a greater area and aspect ratio, suggesting that vinculin structures on our lam-111 LB-film should be smaller and less elongated since the elasticity gradually decreases. Unlike FA area, the values of FA aspect ratio are relatively higher at 1 and 3 mm of the gradient compared to the rest, generally supporting this conclusion. Although the FA aspect ratio at 9 mm does not precisely follow the assumed decreasing trend, its proximity to the value at 7 mm can be regarded as acceptable. Evidence presented by Nathan and colleagues (36) suggests that an increase in integrin binding sites leads to larger FA area. Consequently, the increased laminin density promotes the size of vinculin. This observation suggests that while protein density can enhance FA area, it may limit its elongation. Thus, this experiment indicates that biological cues primarily govern the FA area of cells, with the influence of physical cues, such as stiffness, becoming more pronounced after 7 mm. Additionally, the laminin density appears to have no significant effect on the elongation of the FA.The same staining procedures were also applied to HaCaT cells, and the confocal fluorescence images are shown in Fig. S7. In comparison to hADSCs, vinculin tends to form larger aggregations at the tips of protrusions rather than elongated rods. Additionally, there are fewer actin fibers with alignments in the cytoskeleton. Due to the inability to recognize vinculin aggregations through particle analysis processing, development of a new method is required for the quantification of FAs in HaCaT cells.

### Correlation analysis

Having created substrates with a continuous and known variation of the protein density, we can identify correlations between the different observables. In that way, the analysis of a single substrate can replace any number of experiments, depending on the chosen resolution. To do so, linear, semilogarithmic and double logarithmic plots of two or three properties were created, and linear fits were carried out, returning the Pearson correlation coefficient (R) (see Fig. S8).

We expected the fluorescence signal to be proportional to the calculated amount of deposited protein, which increases hyperbolically toward the end of the gradient ($∼\frac{1}{A_{\mathrm{trough}}})$. However, this is clearly not the case. The linear correlation is very weak (*R* = 0.67). A logarithmic dependence returns a better correlation (Pearson *R* = 0.8), which becomes excellent (*R* = 0.95; slope = 0.1) when the point at the lowest protein density is omitted. The same is true for the stiffness, which has a very strong logarithmic correlation with the protein density (Pearson *R* = −0.99; slope = −0.6) if the first point is omitted (Fig. S8b). That the low protein density part of the substrate is an outlier points either a strong influence of the substrate or an incomplete LB transfer of the protein layer at low density. Incomplete transfer is indeed a common problem for LB transfer at low surface pressure, but as the films appeared to be continuous in AFM images, we suggest that the former is more likely. The low dependence of the fluorescence signal on protein density implies that only a small fraction of the molecules is available to react with the dye, e.g. the top surface area, which does not dramatically increase by adding further molecules. Aggregation, as shown in Fig. 5, may also render a large fraction of the potential binding sites inaccessible. Interestingly, there is a strong linear correlation (*R* = −0.98) between the fluorescence intensity and the stiffness experienced by the AFM tip (Fig. S8c). This observation is in line with our hypothesis that the “free” parts of the molecules are mainly responsible for reducing the stiffness felt by the AFM tip. The calculated protein density, layer stiffness and fluorescence intensity increase monotonously whereas hADSC single-cell adhesion force and cell density display a maximum around the semi-dilute part of the gradient. Therefore, a monotonous correlation between the cellular response and any of the other three properties cannot exist. There is also no monotonous relationship between the hADSC single-cell adhesion force and the number of adherent cells because the latter decreases much stronger towards the end of the gradient than the former (linear *R* = 0.34; double logarithmic *R* = 0.4). That means that either the adherent cell density does not respond monotonously to cell adhesion force or the negative response towards the end is linked to another property. Here, the stiffness would be a natural assumption, as it is the only property that decreases along the gradient. A good correlation exists from 1st to 5th mm between adhesion force and adherent cell density (linear *R* = 0.84; semilog *R* = 0.89) and from 5th to 9th mm between adherent cell density and substrate stiffness (linear *R* = 0.91; semilog = 0.94). The dependence of cell density on substrate stiffness in the second half of the gradient is greater than on adhesion force in the first half (Fig. S8a). This trend is also maintained for HaCaT cells, which show a pronounced negative response to the decreasing film stiffness towards the end of the gradient. Given that the stiffnesses of the soft hydrogels that are typically used in mechanobiology have Young's moduli on the kPa scale (37), one could deem it unlikely that a stiffness gradient in the 10 MPa/mm range should have such a pronounced effect on the number of adherent cells. Stiffnesses in the range of 50–80 MPa, which is where we observed the highest cell density, are on the level of individual collagen fibrils in tendon (38). However, the stiffness of any tissue depends dramatically on the length scale and the deformation mode at which it is probed (39). All loadbearing tissues have a fibrillar substructure, where the tensile modulus of the individual fibrils is typically in the GPa range (26, 40). Combining these loadbearing structures with other materials such as water gives lower tensile moduli in the 10 or 100 MPa range for the tissue as a whole. In contrast, the moduli determined by indentation are typically a few orders of magnitude lower, as they probe a different deformation mode. When cells interact with their environment through FAs, they attach to individual fibrils and pull on them to create traction. Thus, one might argue that cells mainly probe the tensile moduli of the loadbearing structures in their environment, which are greater than the elastic moduli determined by (nano)indentation. It is therefore also not surprising that stem cells migrate and orient towards stiff surfaces in durotaxis experiments (41), and that cells were also found to respond to stiffness gradients in the MPa/mm range (42). Durotaxis of hADSCs could therefore explain why cells are clustered at a gradient length where the cell adhesion force is below the maximum value.

## Conclusion

By using the LB technique, we were able to fabricate Lam-111 density gradient films. Along the depositing direction, the thickness of the film, measured in PBS buffer, increased continuously from 1.9 to 7.5 nm, in line with a continuous increase in intensity upon chemical attachment of a fluorescent dye, while the stiffness decreased monotonously from 231 to 29 MPa. In contrast, the adhesion force between a single hADSC and the gradient film, as well as the number of adherent hADSCs and HaCaT cells, were found to exhibit a maximum around the semi-dilute part of the film. The quantification of the adhesion force and FA area reveals that the adhesion behavior of hADSCs on this film is initially determined by the biological cue (laminin density) and after reaching a peak value at 7 mm, the impact of a physical cue (stiffness) becomes dominant. These results prove that the LB technique can be a robust way to produce 2.5D biomaterials, opening a pathway to assess the nontrivial correlation between variables such as protein density, surface stiffness and cell density. An open question is whether remodeling of the laminin by the cells affects their own behavior, which could be elucidated by comparing with films that were chemically linked to the underlying substrate. In further studies, these gradients could be combined with nano- and microstructured surfaces to introduce topology as a further layer of information, while the application of soft substrates like silicone gels would enable to shift the stiffness gradients to mimic more compliant tissues. Nevertheless, the ultrathin film has limited impact on the cell behavior since in vitro the ECM substrate is um scale. Furthermore, we propose that gradient materials based on Lam-111 and other ECM proteins offer a promising approach for investigating further aspects of cellular behavior, including differentiation and migration.

## Materials and methods

The following procedures are described in the supporting information:

Quantification of Lam-111 adsorption at the air–water interface; fabrication of gradient Lam-111 LB-films; AFM morphology and thickness measurements; QI of gradient film; single-cell adhesion force measurements; cell distribution assay; quantification of FA of hADSCs; fluorescent labeling of Lam-111; isolation and characterization of hADSC; water contact angle measurement; AFM roughness analysis; laminin gradient stability assessment; digital microscopy; statistical and correlation analysis.

## Supplementary Material

pgae202_Supplementary_Data

## Data Availability

The original data generated for this study can be accessed on zenodo using the following DOI: 10.5281/zenodo.10404885.
