# Supplementary material for: Continuous protein-density gradients: A new approach to correlate physical cues with cell response
Source: PNAS Nexus. 2024 May 21;3(6):pgae202. doi: 10.1093/pnasnexus/pgae202 (PMC11152205; doi:10.1093/pnasnexus/pgae202)
Supplement: pgae202_Supplementary_Data [file pgae202_supplementary_data.zip › PNASNEXUS-PNASNEXUS-2023-01365-TR-s02.docx]

[

**Supporting Information for**

Continuous Protein-Density gradients: A new approach to correlate physical cues with cell response

Shanshan Zhang^1,2^, Oliver Felthaus^3^, Lukas Prantl^3^, Nan Ma^1,4^, Rainhard Machatschek^2^*

1: Institute of Chemistry and Biochemistry, Free University of Berlin, 14195 Berlin, Germany

2: Helmholtz Zentrum Hereon, Institute of Active Polymers, 14513 Teltow, Germany

3: Department of Plastic Surgery, University Hospital Regensburg, 93053 Regensburg, Germany

4: Helmholtz Zentrum Hereon, Institute of Sustainable Materials, 14513 Teltow, Germany

* Corresponding author: Rainhard Machatschek

**Email:** rainhard.machatschek@hereon.de

**This PDF file includes:**

Supplementary Materials and Methods

Figures S1 to S8

Tables S1 to S3

**Other supporting materials for this manuscript include the following:**

Movie S1

**Supplementary Materials and Methods**

- 1. **Digital microscopy**

An overview of the gradient films was recorded using a digital microscope DVM6 (Leica, Germany) with PlanAPO FOV12.55 objective in the magnification of 33× under bright field illumination. The images were digitally fused by the software LAS X.

**1.2. Water contact angle measurement**

The wettability of the surface of the gradient film, as well as bare Si wafers, were characterized by the sessile drop method using a drop shape analyzer (DSA 100, Krüss, Hamburg, Germany). Three water droplets with a volume of 2 µL were sequentially attached at the dilute, semi-dilute, and concentrated parts of the film along the gradient direction. The images of the droplets were recorded by the inbuilt camera. Then, the water contact angle of each droplet was calculated via ImageJ with the “Contact Angle” plug-in. Each sample was measured three times.

**1.3. AFM roughness analysis**

1x1 µm^2^; 5 x 5 µm^2^ and 10 x 10 µm^2^ AFM topography images were flattened using line levelling with 1^st^ order polynomials while ignoring outliers using the Gwyddion software. Then, the images were leveled using a 3 point plane. Afterwards, the avg RMS roughness of all scan lines was read out.

**1.4. Fluorescent labeling of Lam-111**

NHS-fluorescein (Pierce, Thermo Fisher) was stored at -20 °C. The containers were opened after equilibrating to room temperature to avoid moisture condensation. 2.5 mg/mL NHS-fluorescein stock solution was prepared in a DMF (99.8%, Alfa Aesar) / ethanol (99.9%, Roth) mixture (v/v = 1:4). The gradient films were placed in a 24-well plate, with each one being immersed in 0.5 mL of diluted NHS-fluorescein solution (1wt % of stock in PBS buffer). After 30 min, the films were washed 3 times with PBS buffer and then scanned at 488 nm with a confocal laser scanning microscope (LSM780, Carl Zeiss, Jena, Germany). The images were taken in partial scan mode with z-Stack and merged into one high dynamic range topography map. 6 zones with a size of 0.3 mm×0.3 mm at the 1^st^, 3^rd^, 5^th^, 7^th^ and 9^th^ mm of the gradient film each were measured (30 zones in total). The fluorescence intensity of each zone was measured as mean gray value using ImageJ.

- 1. **Single-cell adhesion force measurement with HaCaT cells**

HaCaT cells (ATCC, USA), namely, immortalized human epidermal keratinocytes, used for adhesion force measurements were cultured in Dulbecco’s modified Eagle medium(Gibco), supplemented with 10 (v/v) % fetal bovine serum (Sigma), and 1 (v/v) % penicillin-streptomycin (Gibco) at 37 °C and 5 (v/v) % CO_2_. The cells were fed every 2 days and passaged at a ratio of 1 : 3 when cell confluency reached 80%. Right before the experiment, cells were harvested from the culture plate with 0.25 wt % trypsin (Gibco) for 10 min and suspended in Dulbecco’s Modified Eagle Medium with 25 mM HEPES (Gibco), which is CO_2_ independent. To improve the attachment of the tip to the cell, the tip was incubated in 10 ug/mL fibronectin solution (Thermo Fisher Scientific) and the petri dish (6 cm in diameter, TPP, Swiss) was incubated in 3 wt % BSA (MACS) overnight at 4 °C. The The fibronectin-coated tipless cantilever (TL-FM-10, NanoAndMore, Germany) was used to capture a single cell from the cell suspension by pressing the cantilever onto the target cell at a setpoint of 2 nN for 5 s and then lifting the cantilever. When the cell was successfully attached, the measurements of the adhesive force between this single cell and the gradient Lam- 111 film were performed on a JPK AFM CellHesion 200 module at 37 °C. The cantilever was set to approach the surface with a force of 5 nN, stay for 5 s, and retract from the set force value. The detection was firstly conducted at 1 mm of the gradient with 5 measurements and then continued towards the end in 2 mm steps. The differential between the baseline and the minimum detachment force in the curve recorded during retract was defined as the adhesion force(in absolute value). This experiment was repeated three times with three individual samples.

**1.6. Laminin gradient stability assessment**

Lam-111 coated gradient films and gradient films seeded with hADSCs and HaCaT cells for 12 h and 24 h were fixed with PFA. Samples with cells were further permeablized. Then these samples were blocked for 1h in 2 % goat serum at room temperature, followed by incubation in rabbit anti-Laminin beta1 polyclonal antibody (Invitrogen, at a dilution of 1:400 in 1 wt% BSA solution, ThermoFisher Scientific) at 4 °C overnight. After being washed 3 times with PBS, the samples were immersed in goat anti-rabbit secondary antiboy Alexa Fluor 555(Invitrogen, at a dilution of 1:1000 in PBS, ThermoFisher Scientific) for 1 h at room temperature in the dark. Later, the samples were washed three times with PBS. The Lam-111 gradient films were ready to use while the samples with cells were further stained with Alexa Fluor Plus 647 Phalloidin DAPI) (at a dilution of 1 : 1000 in PBS, Thermo Fisher Scientific). Finally, the images of these samples were taken at 1^st^, 3^rd^, 5^th^, 7^th^ and 9^th^ mm of the substrates with confocal fluorescence microscopy using Plan-Apochromat 20×/0.8 as objective.

**1.7. Cell distribution assay of HaCaT cells**

Fused Silica with a size of 10 mm×10 mm were used as substrates for the gradient Lam-111 film. Subsequently, three parallel gradient films and one bare Fused Si wafer were placed in 12-well plates and pre-treated by immersion in PBS-diluted 10 (v/v) % penicillin-streptomycin solution for 30 minutes to ensure sterilization. Then, the HaCaT cells were seeded on these samples at a density of 10^4^ /cm^2^ and were cultured in an incubator at 37 °C with 5 (v/v) % CO_2_. After 14 hours, the cells were fixed with 4.2 wt % formaldehyde (BD Biosciences) for 10 min and washed 3 times with PBS. Next, the samples were treated with 0.5 (v/v) % Triton X-100 (Sigma Aldrich). After that, the cells were labeled with Alexa Fluor Plus 647 Phalloidin (at a dilution of 1:1000 in 1 wt % BSA solution, Thermo Fisher Scientific) for 50 min. This was followed by a 10-minute incubation with 4’,6-diamidino-2-phenylindole (DAPI) (at a dilution of 1:1000 in PBS, Thermo Fisher Scientific) at room temperature in the dark. In the end, the samples were washed 3 times.

The whole substrates were imaged in Tiles-scan mode with z-stack using confocal fluorescence microscopy. The cell distribution on the substrates was analyzed by quantifying the number of DAPI-stained nuclei per unit area using Image J. In the case of gradient samples, the images were subdivided into 9 zones from low protein density to high protein density with a size of 1 mm × 0.8 mm. The experiment was repeated 2 times so that 6 gradient films and 2 controls were analyzed.

**1.8. Isolation and Characterization of hADSC**

Human ADSCs were isolated from lipoaspirate after liposuction of subcutaneous adipose tissue. The Ethics Committee of the University Hospital of Regensburg (Germany) approved of the collection of lipoaspirate and cell isolation after patient’s informed consent (08/117). For liposuction, a 0.9% (w/v) solution of sodium chloride containing adrenaline (1:200,000) was infiltrated using a 2.5-mm injection cannula (Human Med AG, Schwerin, Germany). Subsequently, liposuction was accomplished using a water jet with an even negative pressure of less than 0.5 mbar (Body-Jet, Human Med AG) and 3.8-mm cannulas (Human Med AG). For cell isolation, the harvested lipoaspirate was digested enzymatically. Briefly, 15 ml adipose tissue was mixed with 15 ml alpha MEM (Sigma Alrich, St. Louis, MO, USA) and 150 µl of a 100 U/ml collagenase (from Clostridium histolyticum) in PBS solution (both Sigma Aldrich). The mixture was incubated for 45 min at 37 °C. After filtering (100-µm filter, Merck-Millipore, Billerica, MA, USA) and centrifugation (500 rcf for 5 min) the supernatant was discarded and the cell pellet was resuspended in cell culture medium (α-MEM containing 1 0% heat-inactivated FBS (Pan-Biotech, Aidenbach, Germany), 100 U/ml penicillin, and 100 µg/ml streptomycin (both Sigma-Aldrich). Resuspended cells containing the stromal vascular fraction were seeded into cell culture flasks and allowed to adhere at 37 °C and 5 % CO2 in a humidified atmosphere. Flasks were washed every day to remove non-adherent cells. After three days, cells were washed with PBS and harvested using a Trypsin/EDTA (Promo-Cell) solution for 5 min at 37 °C, centrifuged at 300 rcf for 5 minutes, and washed thrice with FACS-buffer (containing 0.01% sodium azide, 0.5% BSA, and 2 nM EDTA, all Sigma Aldrich). The cells were transferred to two FACS tubes and resuspended in 40 µL FACS-buffer supplemented with either 5 µL APC anti-human CD90 Antibody and 5 µL Alexa Fluor® 488 anti-mouse/human CD44 antibody or their respective isotype controls (APC Mouse IgG1, κ Isotype Ctrl (FC) Antibody, Alexa Fluor® 488 Rat IgG2b, κ Isotype Ctrl Antibody, all BioLegend, San Diego, CA, USA). After incubation on ice in the dark for 1 h, 1 mL FACS-buffer was added and the cells were centrifuged. The supernatant was discarded, and cells were resuspended in 500 µL FACS-buffer and measured using the FACS Canto II (BD Biosciences, Heildelberg, Germany). 50,000 events of each sample were recorded.

**1.9. Statistical and Correlation Analysis**

For single-cell adhesion force measurements and cell distribution assay, the data were analyzed with Origin 9.0 using one-way ANOVA with the Bonferroni test. Significant differences were considered when p$\ll0.05$ and marked by the symbol ‘*’. Origin 9.0 was also used to quantify correlations between the different observables and the calculated layer coverage. The layer coverage was calculated from the trough area corresponding to a position on the substrate. Assuming that the initial coverage was 0.5 layers, the coverage at the end of compression (8.3 mm) is 8.9 layers. For 9 mm, the same coverage was assumed. For easier comparison, all properties were normalized by their value at 1 mm. The number of adherent cells at each integer mm position was interpolated linearly from the neighboring zones. The 9 mm position was interpolated linearly from the 7 - 8- and 8 - 9-mm zones. To identify correlations, the observables were plotted as a function of the layer coverage and of each other.


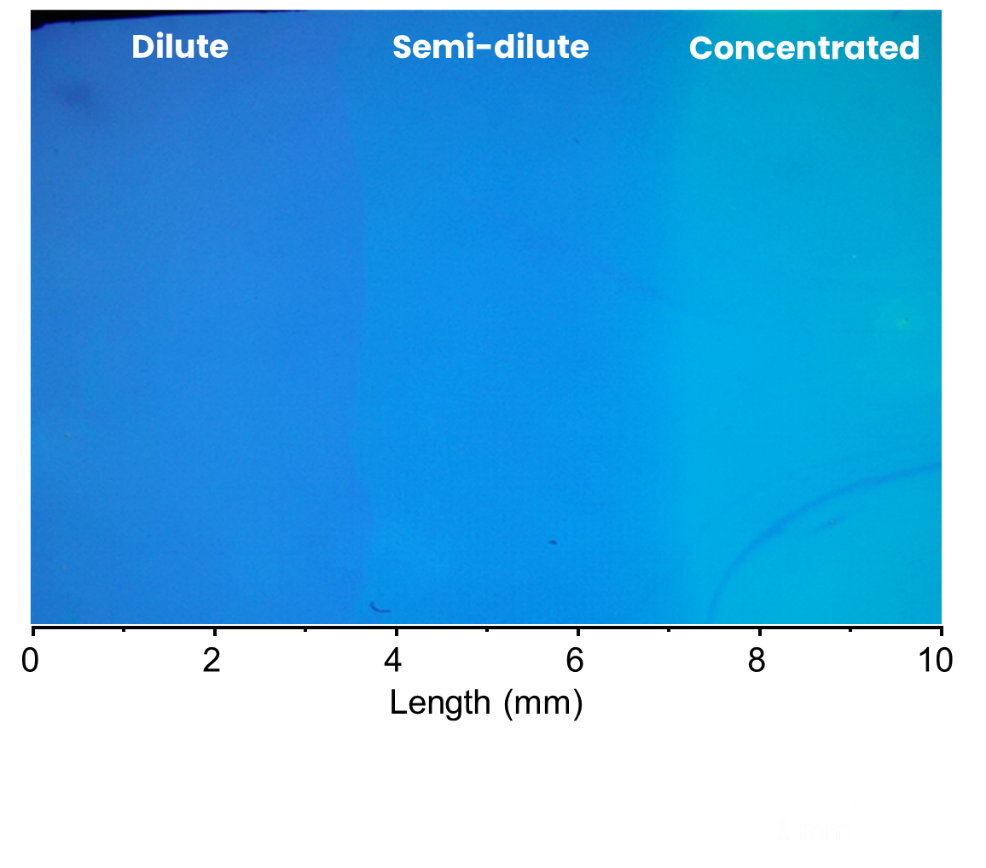


Fig. S1. An optical overview of the Lam-111 LB gradient film on Si wafer. Along the deposition direction, which is from left to right along the axis of substrate length, the intensity of green color enhances. According to the two distinct boundaries, the whole gradient film can be generally divided into three parts, namely dilute, semi-dilute and concentrated. This image can be used as a quick and primary check of the quality of the gradient film.


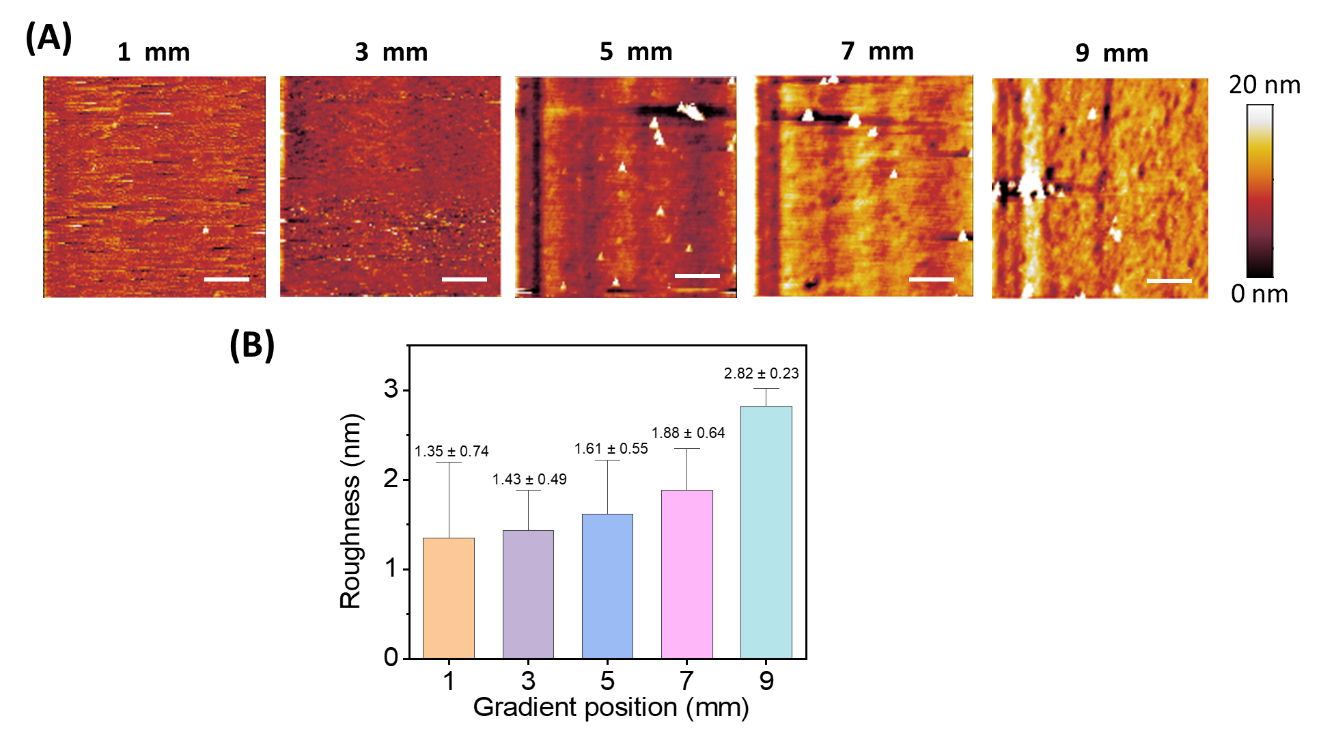


Fig. S2. (A) AFM images of Lam-111 LB gradient film on Si wafer at gradient positions. (B) The results of the RMS Roughness calculated with the AFM images.


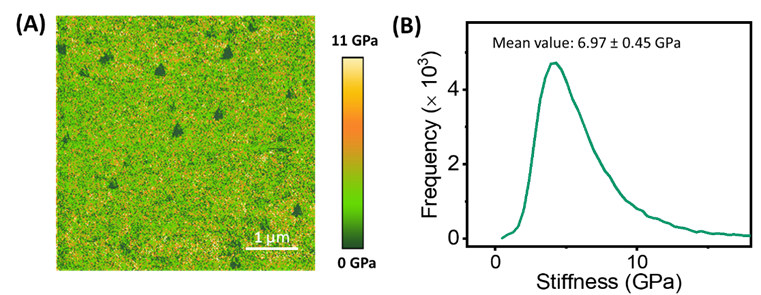


Fig. S3. Results of Si wafer stiffness. (A) Stiffness map of the measured area of bare Si wafer. (B) The corresponding histogram of the stiffness map. The mean value of the stiffness of the bare Si wafer is 6.9 ± 0.1 GPa.


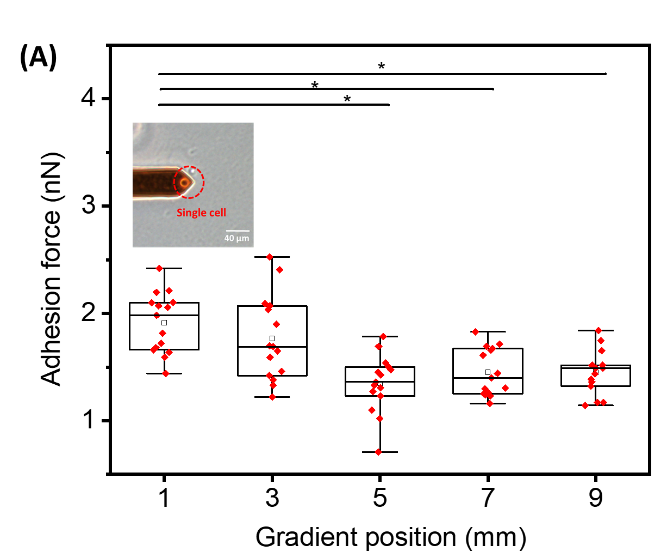


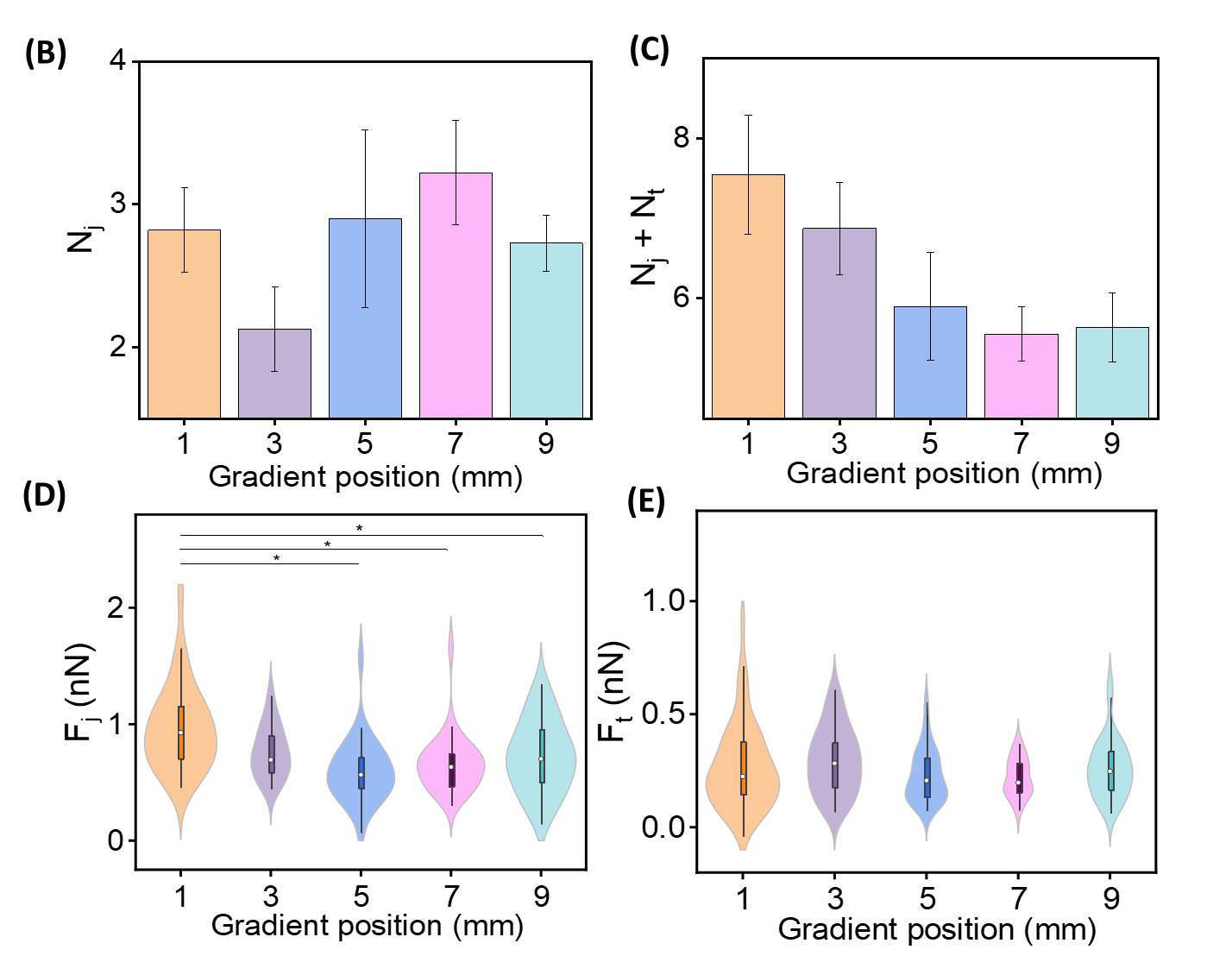


**Fig. S4.** Results of Single-cell spectroscopy with HaCaT cells. (A) Optical microscopy image of a tipless cantilever with a single HaCaT cell attached. (B) The adhesion force between the film and a single HaCaT cell captured by a tipless AFM cantilever was measured at the 1, 3, 5, 7, and 9 mm of the gradient film. The top and bottom of the boxes stand for the 75^th^ and 25^th^ percentile, individually. The middle lines in the boxes stand for the median while the blank cubics stand for the mean value. The upper and lower whiskers indicate the maximum and the minimum. The blue cubes above the boxes are outliers. The significant differences were determined by one-way ANOVA with Bonferroni test, *p≪0.05)


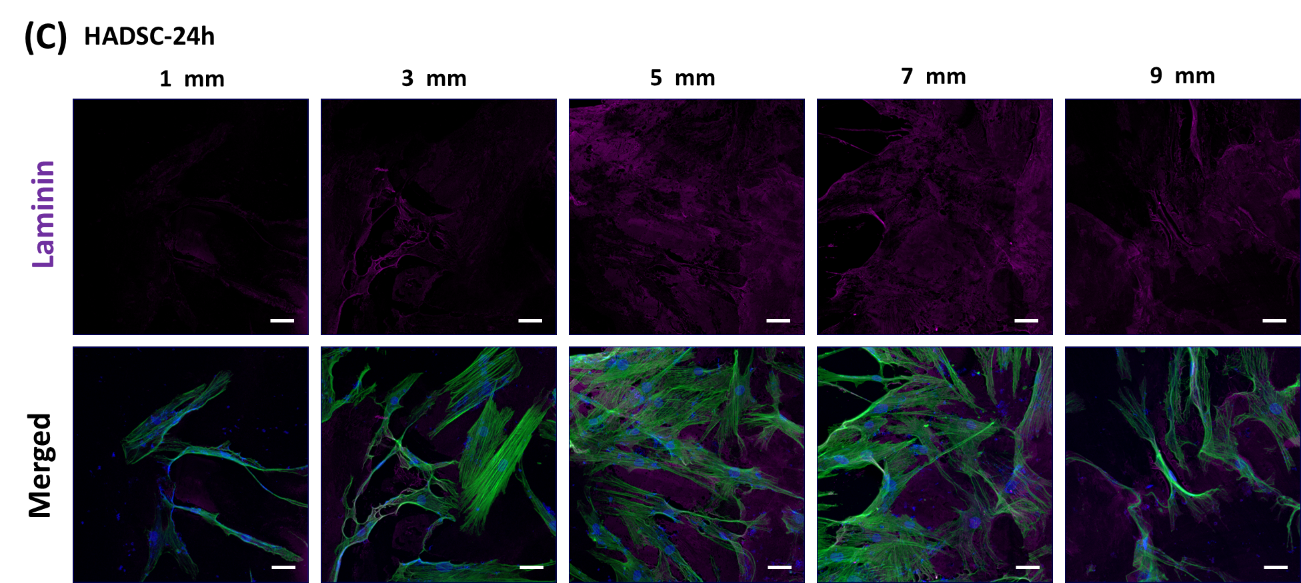

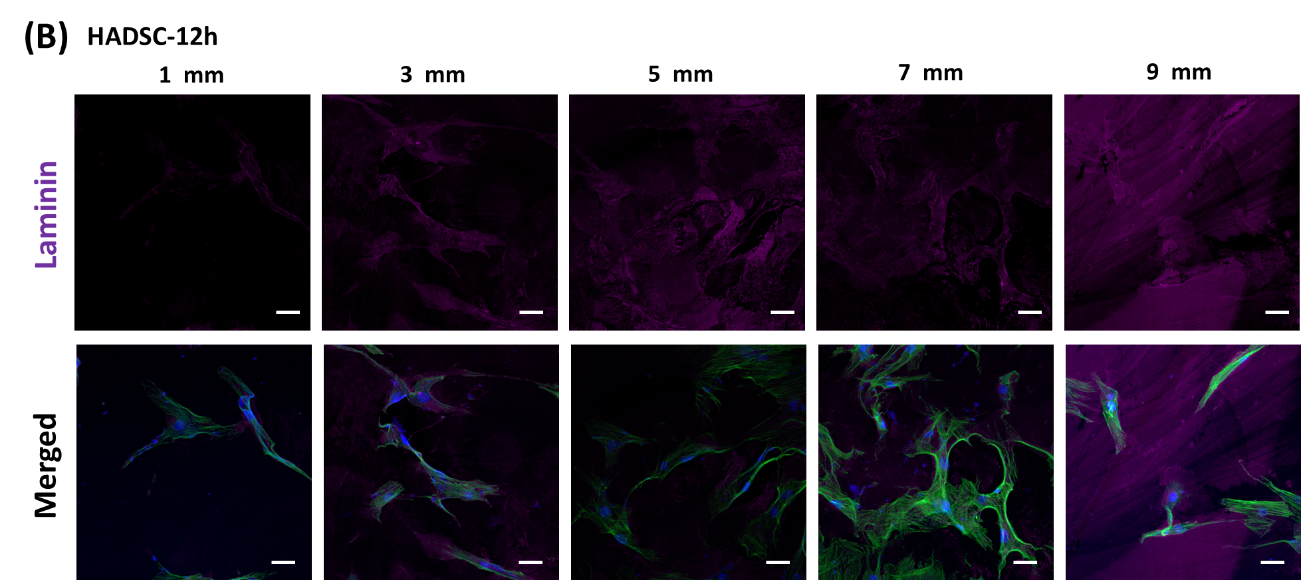

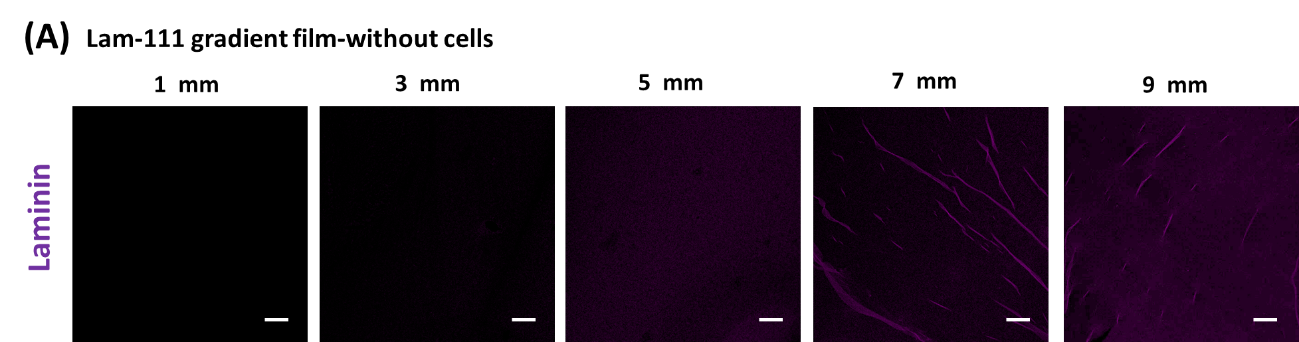


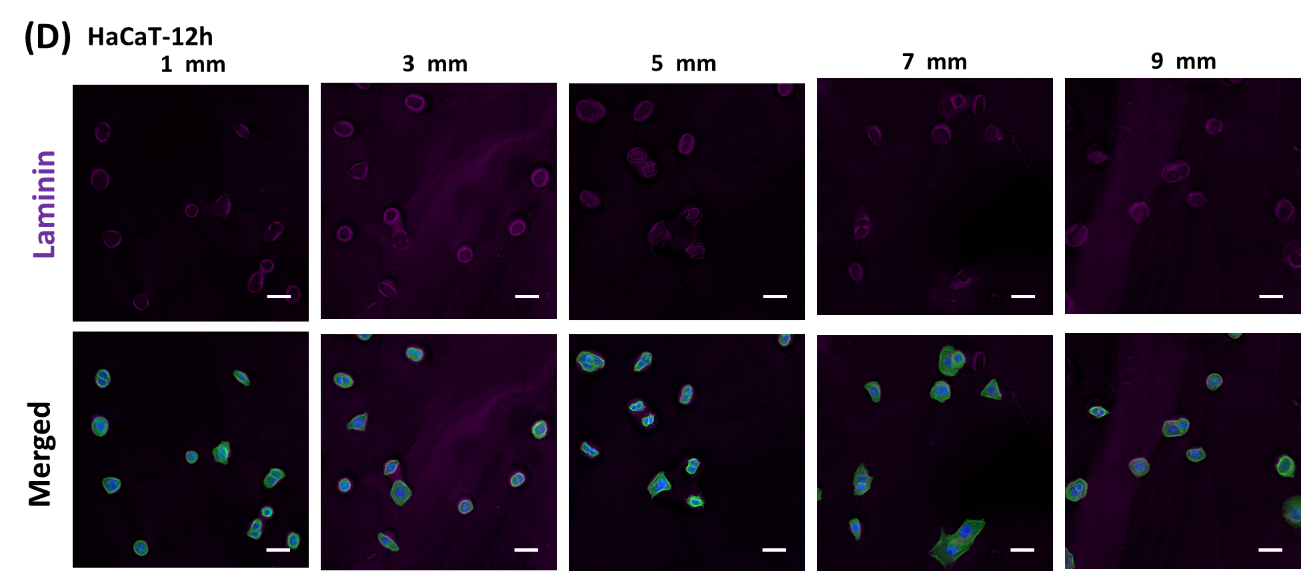


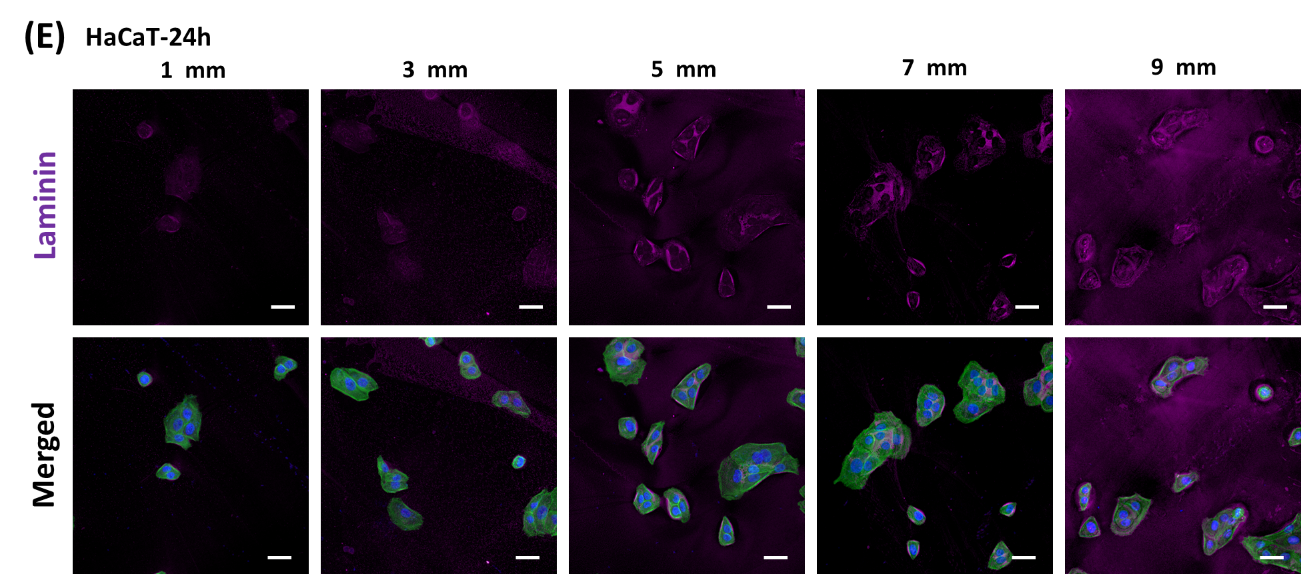


**Fig. S5:** Fluorescence images of laminin gradient and cells on lamnin gradient cultivated for 12h and 24h. Laminin is shown in violet, actin in green and nuclei in blue. Scale bar is 50 µm.


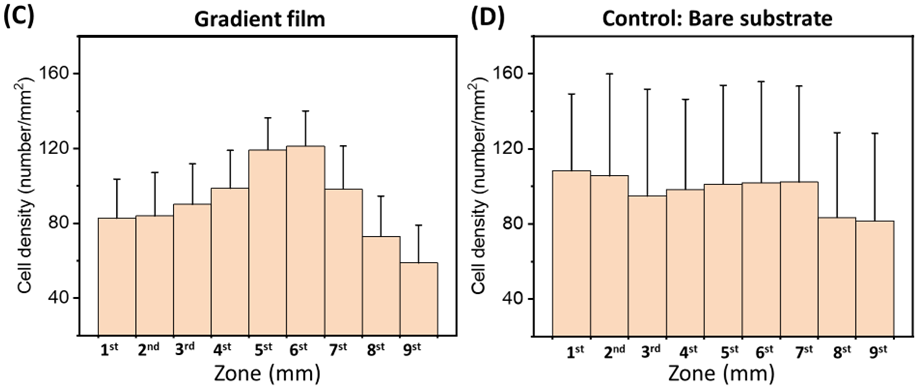

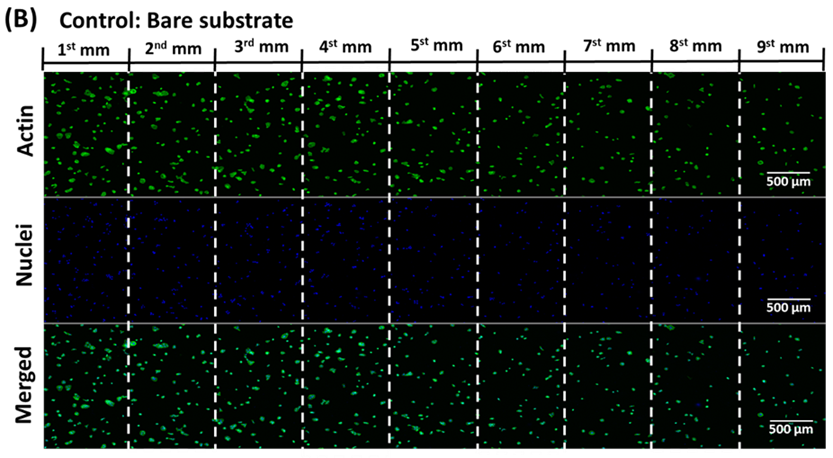

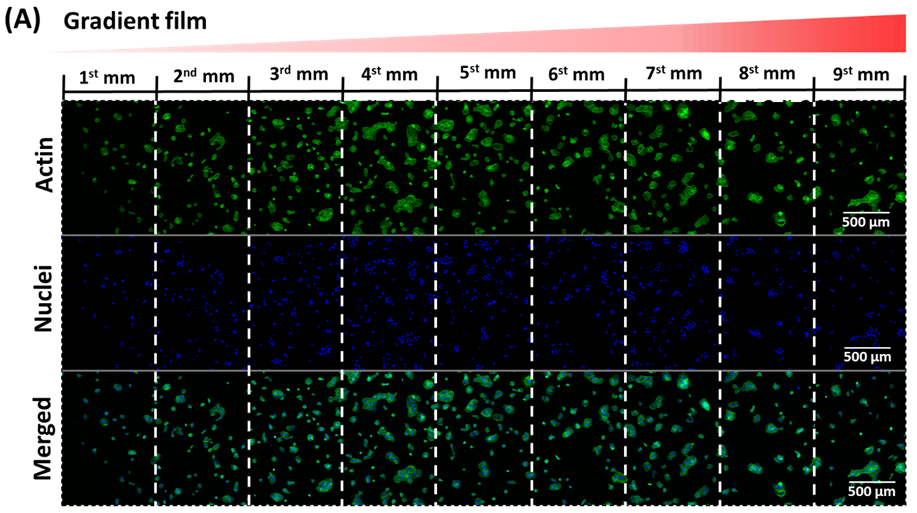


Fig. S6. Cell distribution essay of HaCaT cells. (A) and (B) are Confocal images of HaCaT cells distribution on the gradient Lam-111 film and on bare fused Silica. The cells were fixed and stained after 14 hours of culture at 37 °C, 5 % CO_2_ with a seeding density of 1.0×10^4^ cells/cm^2^. Grun stands for actin and blue stands for the nuclei. (C) and (D) are statistical analyses of the distribution of HaCaT cells on gradient Lamin-111 film and bare substrate. Cell density was calculated by homogeneously dividing the films into 1 mm×8 mm slices. Error bars are the standard error of mean, and significant differences (one-way ANOVA with Tukey's test, n=9, *p<<0.05).


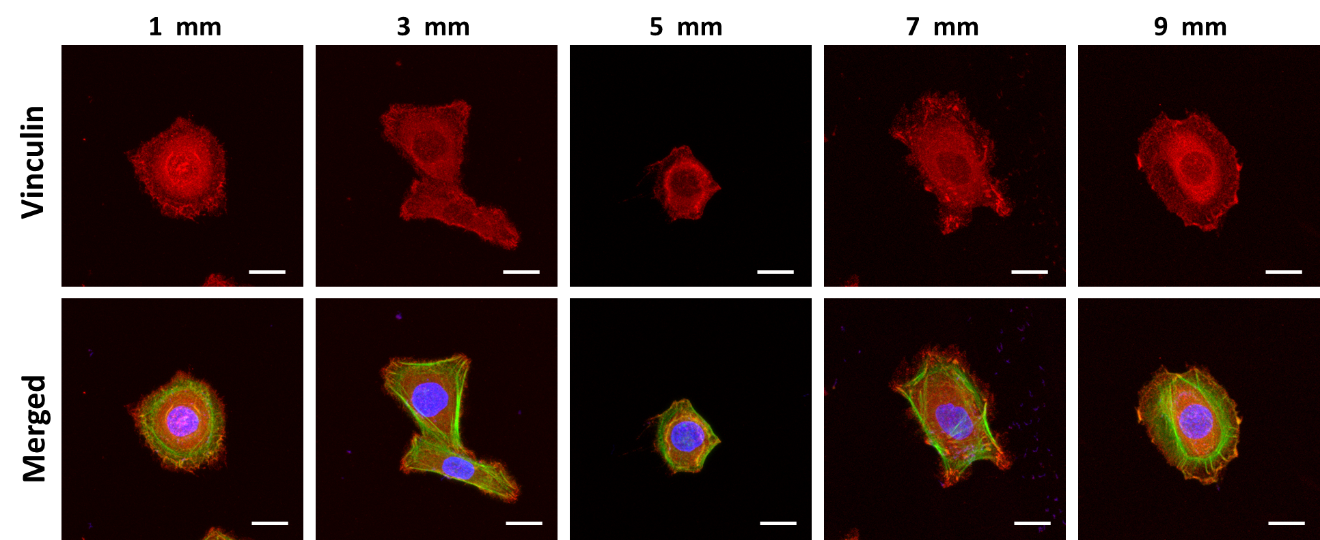

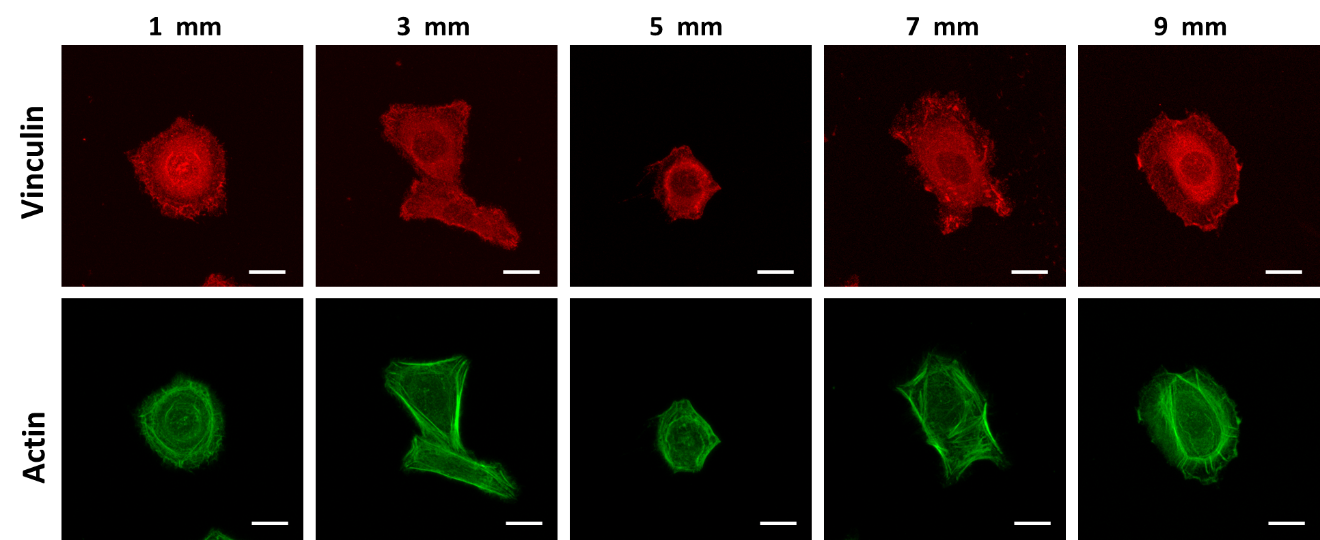


**Fig. S7:** Immunostaining images of the vinculin(red) expression and the corresponding images merged with Phalloidin(green) and nuclei(blue) of HaCaT cultured on the gradient Lam-111 LB film for 12 hours. Scale bar is 20 µm.


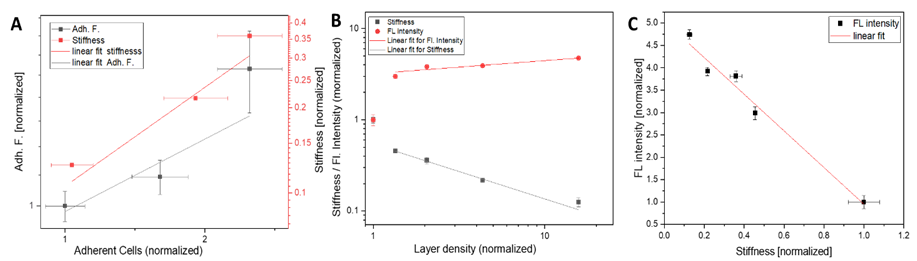


**Fig. S8.** Correlative analysis of observables and calculated protein density. (A) normalized hADSC adhesion force (1mm ; 3 mm; 5 mm) and normalized stiffness (5 mm, 7 mm; 9 mm) vs. normalized number of adherent hADSCs in semilogarithmic scale, together with linear fits. (B) normalized stiffness and normalized fluorescence intensity vs. calculated layer density in logarithmic scale, together with linear fits omitting 1st data point. (C) normalized fluorescence intensity vs. normalized stiffness together with linear fit, and linear scale.

Table S1. The fitting results of the plot of ellipsometric angle Δ vs Lam-111 bulk concentration in the range 0- 0.7 µg/mL.

| Model | Langmuir adsorption |
| --- | --- |
| Equation | y = A*K*x/(1+K*x) |
| Plot | F |
| K | 4.89139 ± 1.72402 |
| A | 4 ± 0.65022 |
| Reduced Chi-Sq | 1104.36751 |
| R-Square (COD) | 0.77159 |
| Adj. R-Square | 0.75957 |

**Table S2.** The mean adhesive force of a single hADSC along the gradient of Lam-111 film.

| **Gradient**  **(mm)** | **Mean**  **(nN)** | **SE**  **(nN)** |
| --- | --- | --- |
| 1 | 1.9 | 0.1 |
| 3 | 2.1 | 0.1 |
| 5 | 3.0 | 0.4 |
| 7 | 4.0 | 0.4 |
| 9 | 3.3 | 0.4 |

**Table S3.** The mean adhesive force of a single HaCaT cell along the gradient of Lam-111 film.

| **Gradient**  **(mm)** | **Mean**  **(nN)** | **SE**  **(nN)** |
| --- | --- | --- |
| 1 | 1.9 | 0.1 |
| 3 | 1.8 | 0.1 |
| 5 | 1.3 | 0.1 |
| 7 | 1.5 | 0.1 |
| 9 | 1.5 | 0.1 |

**Movie S1: The fabrication process of the gradient film on a Langmuir trough.**

The lam-111 solution was already spread on the PBS buffer for 1h. In the movie, two white barriers were moving towards the center at the same rate. Meanwhile, the Si wafer, fixed at the end of the dipping holder, was rising from the subphase at a set speed.
